# A snapshot of Italian nursing homes for people with dementia: A national survey of 1671 facilities

**DOI:** 10.1177/13872877261442226

**Published:** 2026-05-06

**Authors:** Roberta Vaccaro, Patrizia Lorenzini, Francesco Giaquinto, Fabio Matascioli, Emanuela Salvi, Giulia Carnevale, Nicoletta Locuratolo, Nicola Vanacore, Ilaria Bacigalupo, The Permanent Table of the National Dementia Plan Study Group, The NHs Study Group

**Affiliations:** 1 Scientific and Cultural Laboratories, Cognitive Therapy Centre (CTC), Como, Italy; 2 National Centre for Disease Prevention and Health Promotion, Italian National Institute of Health, Rome, Italy; 3 Department of Experimental Medicine, 18976 University of Salento, Lecce, Italy; 4 TAM Onlus, Social Cooperative, San Giorgio a Cremano, Naples, Italy; 5 National Centre for Drug Research and Evaluation, Italian National Institute of Health, Rome, Italy; 6 FONDEM Study Group, Italian National Institute of Health, Rome, Italy

**Keywords:** Alzheimer's disease, dementia, national dementia plan, nursing home, public health, residential services, survey

## Abstract

**Background:**

Nursing homes (NHs) that provide for people with dementia (PWD) are responsible for meeting the needs of patients and their families through medical, personal, and rehabilitative care. However, little is known regarding the Italian facilities.

**Objective:**

To describe the characteristics of NHs and to detail the care services and treatments provided.

**Methods:**

In 2023, the Italian National Institute of Health conducted a national survey to assess dementia care services and update the Dementia Observatory's online Map of Services through a detailed facility questionnaire.

**Results:**

Overall, 1671 NHs accommodating PWD participated, representing 46.3% of facilities and 53.1% of beds. Significant disparities emerged when estimating the number of PWD per available NH across macro-area (North 214; Centre 332; South/Islands 850).

R2D classification was more frequent in southern than central and northern facilities (28%, 10.9%, and 13.1%, respectively; p<0.001). Northern facilities had more rooms (North: 46, Centre: 28, South and Islands: 29; p<0.001) and beds (North: 87, Centre: 55, South and Islands: 58; p<0.001). Southern NHs had shorter admission wait times and longer stays. Conversely, Northern facilities had more staff, better training, and more digital systems and were more often integrated with day care centers and palliative care services.

**Conclusions:**

This is the first national study assessing the accessibility and affordability of Italian NHs housing PWD. The lack of special units, tailored environments and uneven services distribution and staff highlight the fragmentation of Italy's long-term care system. NH geographical distribution is inconsistent with epidemiological estimates of PWD.

## Introduction

The World Health Organization's objectives include the enhancement of healthcare provisions for healthy ageing, with a particular emphasis on the establishment of long-term care (LTC) standards for the elderly, especially those with cognitive impairments.^1^ The LTC was defined as 24-h care in nursing homes (NHs), service homes, hospitals, or health centers for more than 90 days or confirmed by a long-term care decision. Dementia, Parkinson's disease, stroke, depressive symptoms, other mental health disorders, hip fracture, and diabetes increased the risk of moving into NHs by at least 50%.^2^ A previous study reported that dementia increases the likelihood of NH placement by three to five times, remaining the strongest independent predictor compared to others, such as difficulties in daily activities or caregiver stress.^3^ The organization of LTC, especially regarding care approaches for people with dementia (PWD), is one of the major public health and health policy challenges in Europe and worldwide.^4^

It is estimated that more than half of people move to a NH within five years after a dementia diagnosis.^5^ European NHs provide care for the majority of older PWD who need LTC,^6–8^ while the pooled prevalence of mild cognitive impairment (MCI) among older adults living in NH is 21.2% according to a systematic review on 53 studies conducted all over the world.^9^ In Taiwan, the prevalence of all-cause dementia among NH residents was 87.1%,^10^ in the United States, most NHs reported a prevalence of residents with Alzheimer's disease dementia or cognitive impairment ranging from 31% to 80%,^11^ whereas in Brazil, the prevalence of dementia and MCI among NH residents was 62.3% and 15.1%, respectively.^10,12^

Facility characteristics, including higher staffing levels and homelike environments that promote autonomy and social integration, influence resident outcomes, leading to better functional status and greater overall well-being.^13^

According to most studies, the main cause of NH admission among PWD is caregiver distress associated with behavioral symptoms.^14,15^ In this context, NHs are expected to address the unmet needs of PWD and their families by providing health and personal care services, psychosocial and rehabilitative treatments such as physical and occupational therapy, as well as cognitive stimulation.^16^ These services and treatments are part of the person-centered care model in the healthcare settings, previously defined as based on four key attributes: holistic, i.e., care address physical, emotional, social, and spiritual needs, rather than focusing solely on disease or symptoms; individualized, i.e., care is tailored to each person's unique preferences, values, life history, and needs; respectful, i.e., interactions support the person's dignity, autonomy, and rights; empowering, i.e., care actively support individuals in participating in decisions about their care, promoting a sense of control and self-determination.^17^

In Italy, data are lacking on the total number of beds available for PWD in residential facilities as well as on the availability of beds in the private facilities operating under agreement with the national health system or in public facilities. To date, no studies in Italy have examined the territorial configuration of residential care provision for PWD. There is an urgent need to collect and update information on Italian NHs, particularly regarding the appropriateness, accessibility, and affordability of NH services for PWD,^18^ to support improvements in national dementia care management. The Italian National Dementia Plan^19^ emphasizes two key actions: mapping available health and social-care services at national and regional levels, and organizing an integrated network of services that ensures accessibility, quality standards, and multidisciplinary care pathways tailored to local needs, while also addressing social inequalities and conditions of vulnerability. There is a need to better understand the characteristics of the Italian NHs, including their organizational aspects, services provided, actual treatments implemented, and residents’ profiles.^20^

In December 2020, the Italian Parliament funded the Italian Fund for Alzheimer's and other dementias. Its main objective is to give new strategies from a public health point of view and to comprehend the management of dementia care in Italy.^21^ One of the fund's goals was to quantify the alignment between the availability of facilities nationwide and the epidemiological estimates of dementia cases, in accordance with the Italian National Dementia Plan.^19^ Among its objectives, the project aims to draw a national map of services based on data collected through surveys of dementia care facilities.^22^ The present survey was conducted in Italian NHs to describe their characteristics, including the physical environment, and recipients’ profiles, workforce and organizational features, the care services and treatments provided. Based on these results, the online map of Italian NHs has been updated.^23^

## Methods

Representatives of all Italian Regions and Autonomous Provinces provided updated lists of NHs located within their territories, including both publicly owned structures and private facilities providing services under agreement with the National Health System. Directors of the NHs were invited to participate in the study through a cover letter sent by email, followed by a phone call in case of non-response. The study protocol was approved by the Ethics Committee of the Italian National Institute of Health (Protocol 0024270; 22 June 2022). The survey was launched in January 2023 and closed in October 2023. The questionnaire was approved by the Permanent Table of the National Dementia Plan*.* Data were collected through an online platform and exported for statistical analyses. A dedicated telephone number was made available to address any questions and clarify potential doubts.

### Variables

The survey questionnaire consisted of two sections: a profile section (referred to 2022) and a data collection form (referred to 2019). Beyond reporting the 2022 structural characteristics, facilities were also requested to document their activity patterns for 2019 by completing the data collection form.

### Profile section

The profile section was designed to collect information on the NH, defined as an LTC setting providing continuous health and social assistance to non-self-sufficient individuals, mainly older adults, who require 24-h nursing care and support for daily living activities.

In this section data were referred to 2022, distinguishing between territorial facilities and hospitals and specifying the nature of the facility, defined as public or private under agreement and collecting the facility level defined according to the Italian Ministry of Health categorization of 2007 (i.e., R1: intensive care for patients requiring vital function support and the clinical scales, as coma, vegetative states or terminal phase of neurodegenerative diseases; R2: extensive care, for patients with high clinical instability requiring significant 24-h medical care, as parenteral nutrition, functional rehabilitation or pressure injuries; R2D: LTC for patients with high clinical instability in dementia, Special Care Unit for patients with dementia; R3: LTC for patients with ongoing physical conditions that require and supervision, as mobility assistance or nutritional services).^24^ This section also collected information on the accommodation of PWD (e.g., Alzheimer Unit). We gathered information also on the number of beds and rooms. Additionally, we examined the physical environment, such as the presence of a series of common areas and the facilities’ approach to meal preparation. Finally, we collected information on the length of time the facility has been in operation.

### Collection form

In the data collection section, it was requested to provide information referring to 2019, the last year before the pandemic, in order to prevent distortion of the data due to the effects of the COVID-19 containment measures on NH residents, such as the visitation ban and the suspension of group activities. The form gathered data on waiting time for NH admission, length of stay, the number of residents cared for in 2019 and the number of residents with dementia.

Moreover, the form collected information on the presence of a coordinator for social and health services, a workforce coordinator and a case manager. This section also included information about: the percentage of the fee paid by the users, the number of facilities within the territorial care network and the types of services they are connected to (i.e., day care centers, home care services, palliative care services), the presence of computerized medical records and of a registry for tracking the residents’ falls and related outcomes, the use of the multidimensional assessment, staff composition and professional training. Finally, information was collected on the activities, interventions, and assistance services provided in NHs, including general and specialist medical care, nursing care, psychological interventions and recreational activities. Data were also gathered on psychosocial, educational and rehabilitative treatments, as well as other services (e.g., the presence of a day care center within the facility).

### Statistical analysis

Several verification checks were performed to ensure the accuracy of the collected data before beginning the analytical phase.

A detailed description of the characteristics of the NHs participating in the survey was performed both overall and by Italian macro-areas (North, Centre, and South and Islands). Geographical macro areas have been defined according to the categorization of the Italian National Statistics Institute (ISTAT).^25^

The descriptive analysis was based on mean and range for continuous variables, or median and interquartile range in case of asymmetric distributions; frequencies were used to describe categorical variables. Comparisons of characteristics among the three Italian macro-areas were performed using the Kruskal-Wallis test for continuous variables and the Chi-square test or Fisher's exact test for categorical variables.

The number of dementia cases per NH was calculated by dividing the estimated number of dementia cases by the total number of NHs in each Region. Regional estimates of dementia cases were obtained by applying the European age- and sex-specific dementia prevalence rates^26^ to the population aged 65 years and older residing in each Italian Region on January 2022, as reported by ISTAT.^27^

A p-value less than 5% was considered statistically significant. All analyses were performed using STATA software, version SE 17 (Stata Corp, College Station, TX, USA).

## Results

### Facilities’ characteristics referring to 2022

**Figure 1.**
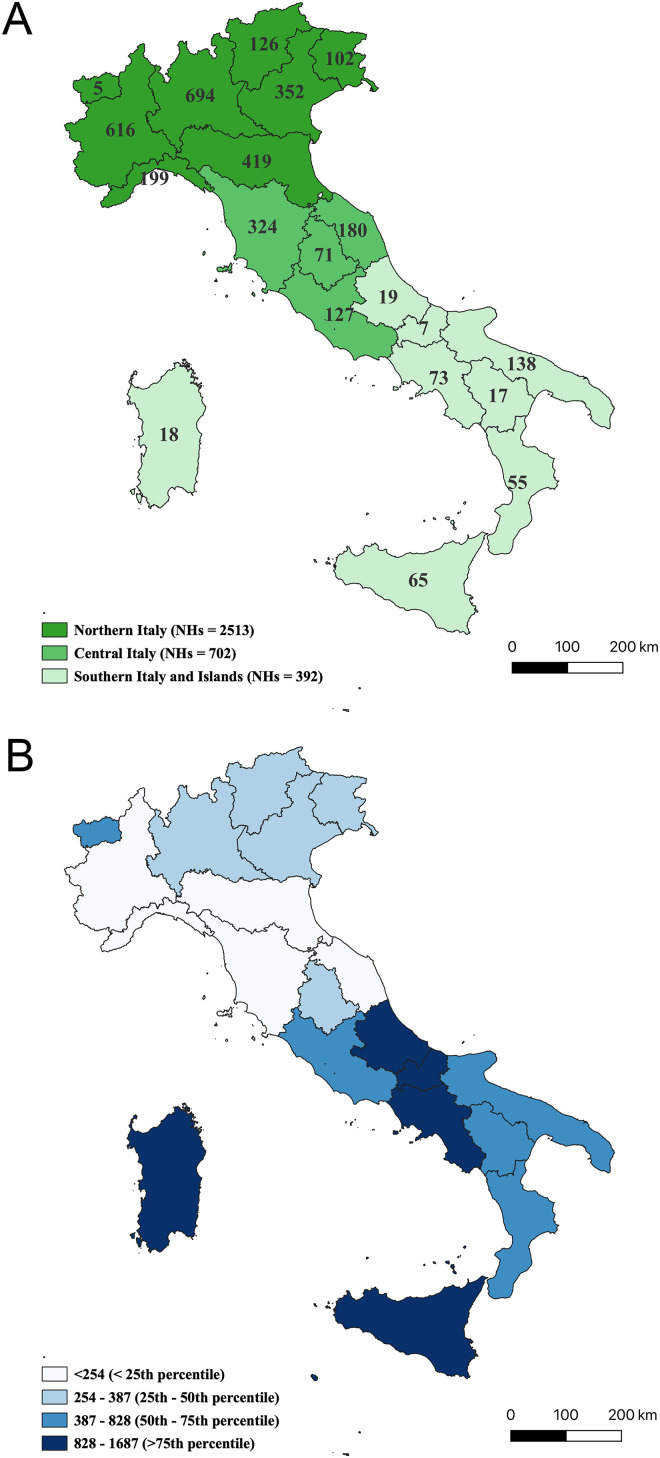
(A) Distribution of the total number of NHs according to Italian macro-area and region, (B) estimated number of dementia cases per NH at the regional level.

For this survey, regional representatives provided the lists of NHs that included a total of 3607 facilities with 243,312 beds. Of them, 1671 facilities accommodating PWD participated in the survey, resulting in a response rate of 46.3%, varying from 52.4% in the Central macro-area to 46.0% in the North and 37.2% in the South and Islands (Supplemental Table 1, Figure 1A). The participating facilities accounted for 129.168 beds, corresponding to 53.1% of the total. A comparison between the 1671 participating NHs and 1936 non-participating ones showed that the former had a higher average number of beds (75 versus 55, p < 0.001) and were more frequently located in the Central area (22% versus 17%, p = 0.005) than in the South (9% versus 13%, p < 0.001).

Among the 1671 participating facilities, a subgroup of 1109, besides providing data on the structural characteristics relative to 2022, also completed the data collection form regarding the NH activities in 2019. Compared with the remaining 562, these 1109 facilities had a higher mean number of beds (83 versus 67, p < 0.001), were more often classified as R2D level (16.2% versus 9.4%, p < 0.001), and were slightly more often private under agreement (95.6% versus 93%, p = 0.049). The geographical distribution was similar between the two groups.

The NHs participating in the survey have been included in the Map of Services on the Dementia Observatory website.^23^ The distribution of NHs across the national territory and the distribution of estimated dementia cases per facility showed significant disparities between geographical macro-areas (Supplemental Table 1, Figure 1A, B).

The estimated number of dementia cases in the North (536.739) was more than twice that of the Centre (232.719) and almost double that of the South (333.369). At the same time, the availability of NHs differed markedly across the three macro-areas: the total number of facilities in the North (2513) was more than six times that in the South and Islands (392) and more than three times that in the Centre (702). Consequently, the estimated number of people with dementia potentially served by each facility was 214 in the North, 332 in the Centre, and increased to 850 in the South and Islands, nearly four times the value observed in the North.

In 2022, NHs classified as R2D represented less than one-seventh of all facilities and were significantly more frequent in the South. Most participating NHs had a territorial setting (92.3%) and were private under agreement (94.7%) (Table 1), with the latter predominantly located in the northern regions (97.1% versus 87.8% for Centre and 93.2% for South). Regarding accommodation arrangements of the guests, in southern Italy, PWD were more often housed in dedicated rooms (28.1% versus 13.1% of North and 16.8% of Centre) or in specific units such as Alzheimer Units (37.7% versus 25.8% of North and 15.8% of Centre). On the contrary, in the North and Centre, PWD more often shared rooms with individuals affected by other conditions (76.8% of North and 78.3% of Centre versus 48.6% of South) (Table 1). NHs located in the North were larger, with an average of 87 beds and 46 rooms, compared to the Centre and South/Islands, which reported an average of 55 beds and 28 rooms, and 58 beds and 29 rooms, respectively (Table 1). At the national level, the proportion of beds usually occupied by PWD was 43% on average, with similar values across the three macro-areas.

**Table 1.** Characteristics of Italian participating facilities at the national level and by geographical macro-areas in 2022 and 2019. Data are expressed as N (%) or mean (min-max).

**Facilities’ characteristics referred to 2022**	**Italy (n = 1671)**	**North (n = 1157)**	**Centre (n = 368)**	**South/Islands (n = 146)**	***p***
Setting, N (%)					
Territorial	1542 (92.3%)	1074 (92.8%)	338 (91.8%)	130 (89.0%)	0.172
Hospital	22 (1.3%)	13 (1.1%)	4 (1.1%)	5 (3.4%)	
Facility level, N (%)					
R2d	233 (13.9%)	151 (13.1%)	40 (10.9%)	42 (28.8%)	<0.001
R1, R2, R3	1173 (70.2%)	813 (70.3%)	266 (72.3%)	94 (64.4%)	
Nature of the facility, N (%)					
Public	65 (3.9%)	20 (1.7%)	37 (10.1%)	8 (5.5%)	<0.001
Private under agreement	1583 (94.7%)	1124 (97.1%)	323 (87.8%)	136 (93.2%)	
Accommodation of PWD, N (%)					
the facility exclusively accommodates PWD	19 (1.1%)	12 (1.1%)	6 (1.6%)	1 (0.7%)	0.558
PWD are allocated in single rooms within the facility	255 (15.3%)	152 (13.1%)	62 (16.8%)	41 (28.1%)	<0.001
PWD belong to a special unit (e.g., Alzheimer Unit)	411 (24.6%)	298 (25.8%)	58 (15.8%)	55 (37.7%)	<0.001
PWD share the room with people with other pathologies	1247 (74.6%)	888 (76.8%)	288 (78.3%)	71 (48.6%)	<0.001
Number of beds,					
mean (min-max)	77 (5–448)	87 (5–448)	55 (8–239)	58 (13–184)	<0.001
Percentage of beds usually occupied by PWD,	43%	43%	43%	40%	
mean (min-max)	(2%-100%)	(3%-100%)	(3%-100%)	(2%-100%)	0.424
Total number of rooms, mean (min-max)	41 (4–266)	46 (5–266)	28 (4–145)	29 (5–77)	<0.001
Single rooms, mean (min-max)	11 (1–82)	12 (1–82)	8 (1–54)	5 (1–27)	<0.001
Double rooms, mean (min-max)	29 (1–188)	32 (1–188)	19 (1–88)	25 (2–77)	<0.001
Rooms with more than two beds, mean (min-max)	9 (1–65)	9 (1–65)	8 (1–35)	10 (1–36)	0.590
Available spaces within the facility, N (%)					
Garden/terrace/covered garden or terrace	1614 (96.6%)	1131 (97.8%)	345 (93.8%)	138 (94.5%)	<0.001
Library/reading room	1209 (72.3%)	847 (73.2%)	263 (71.5%)	99 (67.8%)	0.355
Space for events/theatre	1325 (79.3%)	948 (81.9%)	256 (69.6%)	121 (82.9%)	<0.001
Herbal tea room/bar	800 (47.9%)	594 (51.3%)	130 (35.3%)	76 (52.1%)	<0.001
Alzheimer's garden	317 (19.0%)	243 (21.0%)	39 (10.6%)	35 (24.0%)	<0.001
Living room	1490 (89.2%)	1033 (89.3%)	329 (89.4%)	128 (87.7%)	0.829
Chapel	1294 (77.4%)	934 (80.7%)	259 (70.4%)	101 (69.2%)	<0.001
Gym	1622 (97.1%)	1143 (98.8%)	333 (90.5%)	146 (100%)	<0.001
Meals prepared within the facility, N (%)	1350 (80.8%)	968 (83.7%)	260 (70.7%)	122 (83.6%)	<0.001
How long the facility has been authorized to operate, years, median (IQR)	15.6 (9–22.2)	16.4 (9.6–22.6)	15.5 (8.9–22.7)	11.4 (6.4–16.6)	<0.001

***Facilities’ characteristics referred to 2019***	**Italy** (n = 1109)	**North** (n = 776)	**Centre** (n = 248)	**South/Islands** (n = 85)	***p***
Waiting time for admission to NH, N (%)					
0–3 months	515 (46.4%)	330 (42.5%)	132 (53.2%)	53 (62.3%)	0.001
3–6 months	163 (14.7%)	124 (16.0%)	33 (13.3%)	6 (7.1%)	
6 + months	129 (11.6%)	95 (12.2%)	21 (8.5%)	13 (15.3%)	
Duration of stay of PWD in the facility, N (%)					
<12 months	107 (9.7%)	59 (7.6%)	31 (12.5%)	17 (20.0%)	<0.001
12–24 months	163 (14.7%)	126 (16.2%)	28 (11.3%)	9 (10.6%)	
24–36 months	113 (10.2%)	86 (11.1%)	21 (8.5%)	6 (7.1%)	
>36 months	383 (34.5%)	248 (32.0%)	99 (39.9%)	36 (42.3%)	
Residents in care in 2019,	122	134	98	78	<0.001
mean (min-max)	(2–1811)	(2–1811)	(5–1071)	(18–360)	
Residents with dementia in care in 2019,	48	54	37	26	<0.001
mean (min-max)	(0–500)	(0–500)	(0–426)	(0–125)	
Percentage of the fee paid by the user, mean	52%	57%	41%	36%	<0.001

Missing values: Setting (107, 6.4%), Facility level (265, 15.9%), Nature of the facility (23, 1.4%), Length of authorization to operate (111, 6.6%), Waiting time to access (302, 27.2%), Duration of stay (343, 30.9%), Residents in care (200, 18.8%), Residents with dementia in care (302, 27.2%). PWD: people with dementia; NH: nursing homes

Concerning the physical environment of the NHs, features such as theatres, bars, gyms, and Alzheimer's gardens were less often available in facilities located in Central Italy. Moreover, in this macro-area, meals were less frequently prepared locally (Table 1).

The NHs with the longest authorization to operate were located in the North and Centre (16.4 and 15.5 years respectively), whereas facilities in the South/Islands showed a significantly shorter duration of activity (11.4 years).

### Facilities’ characteristics referring to 2019

Southern facilities participating in the survey showed shorter waiting times for admission (62% less than 3 months versus 53% in the Centre and 42% in the North) and longer duration of stay for PWD (42% > 36 months versus 40% in the Centre and 32% in the North) (Table 1).

In 2019, the total number of residents and the number of residents with dementia were significantly higher in NHs located in the North (134 and 54, respectively) compared with the Centre (98 and 37) and the South (78 and 26) (Table 1). Approximately half of the fee is borne by residents, with a significantly higher share covered by users residing in Northern Italy (57%) compared with those in the Central (41%) and Southern regions (36%).

### Workforce and organizational features of the Italian NHs referring to 2019

At the national level, most facilities had a coordinator for social and health services (84.2%) and a workforce coordinator (86.7%); these roles were frequently covered by nurses. Conversely, a case manager was present in fewer than half of the facilities (44.7%), and the position was mainly held by a nurse or a physician (Table 2).

**Table 2.** Organizational features of Italian NHs at the national level and by geographical macro-area in 2019. Data are expressed as N (%) or mean (min-max).

**Organizational features**	**Italy (n = 1109)**	**North (n = 776)**	**Centre (n = 248)**	**South/Islands (n = 85)**	***p***
Presence of a general coordinator for social and health services, N (%)	934 (84.2%)	672 (86.6%)	197 (79.4%)	65 (76.5%)	0.017
Type of specialization, N (%)					
Physician*	162 (17.3%)	101 (15.1%)	29 (14.7%)	32 (49.3%)	
Nurse*	451 (48.3%)	323 (48.1%)	111 (56.3%)	17 (26.2%)	
Other*	318 (34.0%)	245 (36.5%)	57 (28.9%)	16 (24.6%)	
Presence of a workforce coordinator, N (%)	961 (86.7%)	669 (86.2%)	222 (89.5%)	70 (82.4%)	0.428
Type of specialization, N (%)					
Physician*	70 (7.3%)	36 (5.3%)	16 (7.3%)	18 (25.7%)	
Nurse*	415 (43.2%)	283 (42.3%)	113 (51.0%)	19 (27.1%)	
Other*	476 (49.5%)	350 (52.3%)	93 (41.9%)	33 (47.1%)	
Presence of a case manager, N (%)	496 (44.7%)	325 (41.9%)	131 (52.8%)	40 (47.1%)	0.023
Type of specialization, N (%)					
Physician*	106 (21.4%)	71 (21.8%)	25 (19.1%)	10 (25.0%)	
Nurse*	184 (37.1%)	109 (33.5%)	69 (52.7%)	6 (15.0%)	
Other*	206 (41.5%)	145 (44.6%)	37 (28.2%)	24 (60.0%)	
The facility is included in the territorial care network, N (%)	761 (68.6%)	538 (69.3%)	173 (69.8%)	50 (58.8%)	0.021
Services the facility is connected to					
CCDD, N (%)	221 (29.0%)	170 (31.6%)	38 (22.0%)	13 (26.0%)	0.047
Daily Care center, N (%)	252 (33.1%)	205 (38.1%)	36 (20.8%)	11 (22.0%)	<0.001
Rehabilitation institute/other residential facilities, N (%)	263 (34.6%)	182 (33.8%)	56 (32.4%)	25 (50.0%)	0.056
Home care/domiciliary assistance provided by NH staff, N (%)	206 (27.1%)	133 (24.7%)	64 (37.0%)	9 (18.0%)	0.002
Palliative care, N (%)	160 (21.0%)	133 (24.7%)	22 (12.7%)	5 (10.0%)	<0.001
Presence of computerized medical records, N (%)	746 (67.3%)	580 (74.7%)	137 (55.2%)	29 (34.1%)	<0.001
Presence of a registry for tracking residents’ falls and related outcomes, N (%)	1004 (90.5%)	717 (92.4%)	215 (86.7%)	72 (84.7%)	<0.001
Multidimensional assessment and periodic follow-ups, N (%)	972 (87.7%)	691 (89.0%)	206 (83.1%)	75 (88.2%)	0.139
Assessment tools adopted, N (%)					
Braden/Norton§	564 (58.0%)	390 (56.4%)	153 (74.3%)	21 (28.0%)	<0.001
IADL§	450 (46.3%)	277 (40.1%)	127 (61.7%)	46 (61.3%)	<0.001
Barthel ADL§	839 (86.3%)	603 (87.3%)	179 (86.9%)	57 (76.0%)	0.025
MMSE§	735 (75.6%)	539 (78.0%)	150 (72.8%)	46 (61.3%)	0.004
NPI§	333 (34.3%)	277 (40.1%)	47 (22.8%)	9 (12.0%)	<0.001
Tinetti§	158 (16.3%)	120 (17.4%)	33 (16.0%)	5 (6.7%)	0.024
Facilities with at least one of the following professionals in the staff, N (%)					
neurologist	110 (9.9%)	58 (7.5%)	27 (10.9%)	25 (29.4%)	<0.001
geriatrician	337 (30.4%)	243 (31.3%)	52 (21.0%)	42 (49.4%)	<0.001
psychiatrist	66 (6.0%)	48 (6.2%)	7 (2.8%)	11 (12.9%)	0.003
psychologist	546 (49.2%)	384 (49.5%)	104 (41.9%)	58 (68.2%)	<0.001
neuropsychologist	12 (1.1%)	9 (1.2%)	2 (0.8%)	1 (1.2%)	0.893
nurse	990 (89.3%)	693 (69.3%)	222 (89.5%)	75 (88.2%)	0.946
social worker	381 (34.4%)	211 (27.2%)	101 (40.7%)	69 (81.2%)	<0.001
physiotherapist	971 (87.6%)	688 (88.7%)	210 (84.7%)	73 (85.9%)	0.226
speech therapist	179 (16.1%)	155 (20.0%)	22 (8.9%)	2 (2.4%)	<0.001
occupational therapist	163 (14.7%)	79 (10.2%)	63 (25.4%)	21 (24.7%)	<0.001
nutritionist	118 (10.6%)	44 (5.7%)	56 (22.6%)	18 (21.2%)	<0.001
educator	581 (52.4%)	426 (54.9%)	102 (41.1%)	53 (62.4%)	<0.001
community coordinator	347 (31.3%)	246 (31.7%)	85 (34.3%)	16 (18.8%)	0.027
social and health care assistant	936 (84.4%)	652 (84.0%)	212 (85.5%)	72 (84.7%)	0.855
psychiatric rehabilitation therapist	30 (2.7%)	18 (2.3%)	8 (3.2%)	4 (4.7%)	0.370
support services staff (cleaning and food)	504 (45.4%)	348 (44.8%)	108 (43.5%)	48 (56.5%)	0.098
Total number of professionals, N (%)					
0–20	181 (16.3%)	111 (14.3%)	60 (24.2%)	10 (1.8%)	<0.001
21–50	482 (43.5%)	324 (41.8%)	109 (44.0%)	49 (57.6%)	
51–100	283 (25.5%)	215 (27.7%)	54 (21.8%)	14 (16.5%)	
100+	76 (6.9%)	67 (8.6%)	7 (2.8%)	2 (2.4%)	
Total hours of staff training per professional, mean (min-max)	16.5 (0–900)	18.7 (0–900)	9.5 (0–210)	16.2 (0–343)	<0.001
Facilities with at least one trained professional, N (%)					
neurologist	27 (2.4%)	9 (1.2%)	10 (4.0%)	8 (9.4%)	<0.001
geriatrician	185 (16.7%)	133 (17.1%)	29 (11.7%)	23 (27.1%)	0.004
psychiatrist	21 (1.9%)	9 (1.2%)	6 (2.4%)	6 (7.1%)	0.001
psychologist	294 (26.5%)	208 (26.8%)	54 (21.8%)	32 (37.6%)	0.016
neuropsychologist	10 (0.9%)	8 (1.0%)	1 (0.4%)	1 (1.2%)	0.636
nurse	931 (83.9%)	663 (85.4%)	202 (81.5%)	66 (77.6%)	0.085
social worker	285 (25.7%)	167 (21.5%)	67 (27.0%)	51 (60.0%)	<0.001
physiotherapist	773 (69.7%)	574 (74.0%)	143 (57.7%)	56 (65.9%)	<0.001
speech therapist	116 (10.5%)	101 (13.0%)	14 (5.6%)	1 (1.2%)	<0.001
occupational therapist	133 (12.0%)	64 (8.2%)	53 (21.4%)	16 (18.8%)	<0.001
nutritionist	34 (3.1%)	13 (1.7%)	18 (7.3%)	3 (3.5%)	<0.001
educator	485 (43.7%)	367 (47.3%)	77 (31.0%)	41 (48.2%)	<0.001
community coordinator	264 (23.8%)	195 (25.1%)	58 (23.4%)	11 (12.9%)	0.043
social and health care assistant	909 (82.0%)	654 (84.3%)	192 (77.4%)	63 (74.1%)	0.007
psychiatric rehabilitation therapist	11 (1.0%)	4 (0.5%)	3 (1.2%)	4 (4.7%)	0.001
support services staff (cleaning and food)	406 (36.6%)	288 (37.1%)	86 (34.7%)	32 (37.6%)	0.770

Missing values: Presence of a social and health services coordinator (14, 1.3%) Specialization of the social and health services coordinator (3, 0.3%), Presence of a workforce coordinator (14, 1.3%), Specialization of the workforce coordinator (0, 0%), Presence of a case manager (21, 1.9%), Specialization of the case manager (2, 0.4%), Inclusion in the territorial care network (99, 8.9%), computerized medical records (14, 1.3%), Registry of tracking residents’ falls (70, 6.3%), Multidimensional assessment (17, 1.5%), Staff data (87, 7.8%), Staff training hours (66, 6.0%).

*Percentage calculated based on the facilities in which the figure is present.

§Percentage calculated based on the facilities that made the evaluation.

ADL: Activities of Daily Living; CCDD: Centers for Cognitive Disorders and Dementias; IADL: Instrumental Activities of Daily Living; MMSE: Mini-Mental State Examination; NH: nursing home; NPI: Neuropsychiatric Inventory.

Facilities in the South were included in a network of territorial services less frequently than those in other areas (58.8% in the South versus 69.3% in the North and 69.8% in the Centre) (Table 2). The network more often included day care centers and palliative care services for facilities in the North, whereas home care assistance was more frequently part of the network in the Centre. The presence of computerized medical records and of a registry for tracking the residents’ falls was significantly more common in the North (92.4% versus 86.7% in the Centre and 84.7% in the South).

In most facilities (87.7%), PWD received an initial multidimensional assessment and periodic follow-ups. Overall, the tools most commonly used were the Barthel Activities of Daily Living (ADL) index^28^ (86.3%), the Mini-Mental State Examination (MMSE)^29^ (75.6%), the Braden scale,^30^ and the Norton scale^31^ (58.0%).

Facilities in the South, compared with those in the Centre and North, more frequently employed medical professionals (neurologists: 29.4% versus 7.5% in the North and 10.9% in the Centre; geriatricians: 49.4% versus 31.3% in the North and 21.0% in the Centre) and psychologists (68.2% versus 49.5% in the North and 41.9% in the Centre), social workers (81.2% versus 27.2% in the North and 40.7% in the Centre) and educators (62.4% versus 54.9% in the North and 41.1% in the Centre). Occupational therapists were more commonly present in NHs in the Centre (25.4%) and South (24.7%) than in the North (10.2%).

Northern facilities had a higher number of care staff compared with those in South and Centre (45 versus 36 versus 31, respectively, data not shown). The yearly training hours per care professional were similar in facilities in the North and South and Islands and higher than in those located in the Centre (Table 2). Considering the ratio between the number of beds and the number of care staff, facilities in the North and Centre showed similar values (2.8 and 2.7, respectively), both significantly higher than the value observed in the South (1.8; p = 0.001) (data not shown).

### Care services and treatments referring to 2019

Regarding care services provided in the facilities, general medical care, nursing services, speech therapy, personal care services, and social and recreational activities were more common in Northern facilities. On the other hand, specialist medical care, psychological support, social assistance, and occupational therapy were more prevalent in Southern NHs (Table 3). In terms of psychosocial and rehabilitative treatments, facilities in the South more frequently offered reality orientation therapy,^32^ reminiscence therapy,^33^ cognitive-behavioral therapy,^34^ sensory gardens,^35^ and dance-movement therapy.^36^ Conversely, Northern NHs more commonly provided doll therapy,^37^ pet therapy,^38^ touch therapy,^34^ and Snoezelen rooms.^39^

**Table 3.** Care services and treatments provided in the facilities at the national level and by geographical macro-area during 2019. Data are expressed as N (%).

**Care services and treatments**	**Italy (n = 1109)**	**North (n = 776)**	**Centre (n = 248)**	**South/Islands (n = 85)**	***p***
*Activities, interventions, and assistance services provided, N (%)*					
General medical care	1035 (93.3%)	749 (96.5%)	207 (83.5%)	79 (92.9%)	<0.001
Specialistic medical care	591 (53.3%)	395 (50.9%)	128 (51.6%)	68 (80.0%)	<0.001
Nursing care	1082 (97.6%)	763 (98.3%)	239 (96.4%)	80 (94.1%)	0.022
Psychological intervention	668 (60.2%)	481 (62.0%)	117 (47.2%)	70 (82.4%)	<0.001
Physiotherapy	1065 (96.0%)	751 (96.8%)	232 (93.5%)	82 (96.5%)	0.075
Social assistance	512 (46.2%)	309 (39.8%)	128 (51.6%)	75 (88.2%)	<0.001
Speech therapy	260 (23.4%)	214 (27.6%)	39 (15.7%)	7 (8.2%)	<0.001
Occupational activities	803 (72.4%)	545 (70.2%)	183 (73.8%)	75 (88.2%)	0.002
Animation, socialization, and recreational activities	1058 (95.4%)	746 (96.1%)	236 (95.2%)	76 (89.4%)	0.019
Podiatry/pedicure/hairdressing/barber services	1032 (93.1%)	742 (95.6%)	223 (89.9%)	67 (78.8%)	<0.001
Nutritionist service	422 (38.1%)	269 (34.7%)	112 (45.2%)	41 (48.2%)	0.002
*Psychosocial, educational and rehabilitation treatments, N (%)*					
Cognitive stimulation	987 (89.0%)	694 (89.4%)	216 (87.1%)	77 (90.6%)	0.526
Reality Orientation Therapy	545 (49.1%)	385 (49.6%)	99 (39.9%)	61 (71.8%)	<0.001
Reminiscence therapy	434 (39.1%)	296 (38.1%)	91 (36.7%)	47 (55.3%)	0.006
Doll therapy	515 (46.4%)	390 (50.3%)	94 (37.9%)	31 (36.5%)	<0.001
Pet therapy	388 (35.0%)	299 (38.5%)	70 (28.2%)	19 (22.4%)	<0.001
Validation therapy	225 (20.3%)	173 (22.3%)	33 (13.3%)	19 (22.4%)	0.008
Conversationalism	509 (45.9%)	349 (45.0%)	116 (46.8%)	44 (51.8%)	0.467
Cognitive-behavioral therapy	564 (50.9%)	379 (48.8%)	121 (48.8%)	64 (75.3%)	<0.001
Touch therapy/massage	320 (28.9%)	254 (32.7%)	49 (19.8%)	17 (20.0%)	<0.001
Shiatsu	7 (0.6%)	7 (0.9%)	0 (0%)	0 (0%)	0.221
Light therapy	22 (2.0%)	16 (2.1%)	2 (0.8%)	4 (4.7%)	0.081
Sensory garden	186 (16.8%)	117 (15.1%)	39 (15.7%)	30 (35.3%)	<0.001
Horticultural therapy	403 (36.3%)	278 (35.8%)	96 (38.7%)	29 (34.1%)	0.646
Music therapy	674 (60.8%)	459 (59.1%)	157 (63.3%)	58 (68.2%)	0.173
Dance Movement Therapy	192 (17.3%)	113 (14.6%)	49 (19.8%)	30 (35.3%)	<0.001
Aromatherapy	174 (15.7%)	125 (16.1%)	35 (14.1%)	14 (16.5%)	0.738
Art therapy	380 (34.3%)	256 (33.0%)	92 (37.1%)	32 (37.6%)	0.392
Snoezelen room	99 (8.9%)	81 (10.4%)	15 (6.0%)	3 (3.5%)	0.021
*Presence of other services*					
Day care center	312 (28.1%)	255 (32.9%)	45 (18.1%)	12 (14.1%)	<0.001
Domiciliary assistance provided by NH staff	256 (23.1%)	212 (27.3%)	32 (12.9%)	12 (14.1%)	<0.001
Respite care	541 (48.8%)	400 (51.5%)	108 (43.6%)	33 (38.8%)	0.017

Missing values: Day care center (10, 0.9%), Domiciliary assistance (17, 1.5%), Respite care (12, 1.1%).

NH: nursing home.

NHs in the northern macro-area more frequently provided domiciliary assistance by their own staff (27.3% versus 12.9% in the Centre and 14.1% in the South), as well as access to day care centers (32.9% versus 18.1% in the Centre and 14.1% in the South) and respite care services (51.5% versus 43.6% in the Centre and 38.8% in the South) (Table 3).

## Discussion

To our knowledge, this is the first Italian comprehensive survey aimed at examining the appropriateness, accessibility and affordability of NHs where PWD live. Due to the absence of a public tracking system, limited information is available on NHs’ structure, services and treatments implemented, and residents’ profiles.^20^

The survey response rate of 46.3%, despite the coordinated efforts between the representatives from the Italian Regions and personnel involved, was lower than expected. Nevertheless, our response rate is consistent with that of 45% obtained in the National Post-acute and Long-term Care Study,^40^ which monitors the provision and use of LTC services in the US, including NHs. This study shares some methodological features with ours, such as the division into geographical macro-areas and some core items of the administered questionnaire (e.g., number of people cared for annually, staff composition and services provided).^41^ European research on LTC facilities reported heterogeneous results on survey participation. Cross-sectional studies conducted in Germany, the Netherlands, and France reported lower response rates (33%, 17%, and 15%, respectively), despite strategies to improve participation, suggesting potential selection bias.^42,43^ Conversely, a UK study on COVID-19 in LTC facilities achieved a higher response rate (53.4%) than ours, likely because the survey aimed to rapidly provide evidence to guide the pandemic response.^44^

Our results on the uneven distribution of NHs across the national territory relative to estimated dementia cases per NH raise equity concerns. While the number of facilities in the North provides relatively adequate coverage of the needs, with 214 estimated PWD per facility, the South and Islands remain far from meeting the demand, with approximately 850 estimated PWD per facility. In addition, Northern facilities showed a substantially higher average number of beds (87) compared with Southern ones (58). However, facilities in the South tended to be more recently established, more specialized and tailored to the needs of PWD, and more frequently staffed with specialized care personnel.

In Italy, a similar pattern of inequality has also been documented for centers dedicated to the diagnosis and care of patients, as well as for day care centers.^22,45^ These differences across Italian macro-areas may partly reflect varying levels of demand associated with major cultural and economic differences across macro-areas. This gap is expected to widen given that, according to the 2021 Ageing Report of the Ageing Working Group, as population longevity increases, the number of dependent older people is expected to rise from about 30.8 million in 2019 to 33.7 million in 2030 and 38.1 million in 2050, representing an overall increase of 23.5%.^46^

Our survey also revealed a higher presence of double rooms in Northern NHs and more shared rooms between PWD and people with other pathologies in both Northern and Central macro-areas compared with the South. This highlights an issue of appropriateness related to environmental crowding, which may exacerbate behavioral and psychological symptoms of dementia (BPSD) and increase mortality risk, as observed during the pandemic.^47^

### Facilities’ characteristics referred to 2022

The residential facilities were mostly private under agreement, especially those located in the Northern regions. This is consistent with the higher socio-economic level of these areas, where private companies tend to invest in the healthcare sector, while in the South, the supply is limited and fragmented, with extensive reliance on informal care work.^48,49^ Increased public expenditure is needed to support social protection, especially for low-income older adults who are more likely to need and less able to afford LTC.^50^

Another important finding is the overall scarcity of facilities with an R2D classification and their greater concentration in the South. These facilities, equipped with Special Care Units for PWD with BPSD, are recommended for patients with clinical instability in dementia^24^ and offer more appropriate care than traditional NHs (i.e., reduced physical restraints, better continence management, decreased use of feeding tubes and fewer hospitalizations).^51^ Furthermore, our finding is close to the 14.9% reported in a previous US study, where the low prevalence highlights a common problem of adequacy of the traditional NHs in terms of staff training in dementia care, and the availability of activities and programs suitable for PWD.^51^ To date, Italy lacks a univocal definition of the residential care recipients, resulting in services targeting individuals with widely varying needs.^52^ As a result, PWD are often located in non-specific units and share rooms with people with other pathologies.

The present survey showed that, in 2022, the average number of beds per NH was substantially higher in the North than in the Centre and South/Islands, reflecting a greater supply in the North. Moreover, slightly more than 40% of the available beds were occupied by PWD, with similar proportions across all geographical macro-areas. This result is lower than what has been reported in studies conducted in European^8,53^ and Asian countries.^10^ One plausible explanation is the extensive reliance in Italy on informal care, with a high proportion of PWD managed at home. However, providing non-specialized care in a non-specialized environment may adversely affect the quality of life of PWD and their families,^54,55^ particularly in the presence of behavioral disturbances.

Our survey also collected information on the characteristics of the built environment of the facilities. We found a limited availability of single rooms in Italian NHs, suggesting undervaluation of privacy in the perceived quality of residential care, although its well-established role as a keystone of the “feeling at home” in NH.^56,57^ Conversely, indoor and outdoor quasi-public spaces, such as gardens, theatres or event areas, tea rooms or bars, gyms, and chapels, were more commonly found in the North and in the South and Islands. However, the Alzheimer's garden, which is beneficial in dementia care for reducing BPSD, residents’ falls and use of antipsychotic medications,^35,58^ is present in only 19% of the facilities. This limited attention to the environment as a key component of a person-centered approach^59^ is surprising, especially considering that the participating NHs are relatively new facilities, with operating authorizations issued between 11.4 years ago in the South and Islands and 16.4 years ago in the North. Nonetheless, there is still no consensus on how to design garden-use interventions in a way that allows for a reliable assessment of their impact.

### Facilities’ characteristics referred to 2019

Regarding the availability of Italian NHs in 2019, our data show that at the national level, in about half of the facilities, the waiting time for admission was under 3 months, while in 26% exceeded 3 months, particularly in those located in the Centre and in the South and Islands. This suggests that demand often exceeds supply, leading to waiting lists for NH admission and increased hospital utilization,^60^ an issue that affects mainly the North. This situation places substantial pressure on LTC systems, a pressure expected to intensify in the coming years as populations continue to age. In response, between 2011 and 2021, most of the 38 member states of the Organization for Economic Co-operation and Development increasingly shifted their focus toward home-based care.^61^

Furthermore, our findings on the percentage of fees paid by users (52% overall, 57% in the North compared with 37% in the Centre and 26% in the South and Islands) highlight significant barriers to the affordability of NH care, in addition to the previously noted challenges in NH availability. Strengthening social protection through increased public expenditure is essential to meet LTC needs, including residential care, as older adults with lower income levels are both more likely to require LTC and less able to afford it.^46^

### Workforce and organizational features of the Italian NHs referred to 2019

As previously noted, Italian NHs lacked national standards for residential services and required professionals. Most facilities had both a general coordinator for social and health services and a workforce coordinator, key roles in linking professionals and overseeing management,^62^ which contribute to improving care quality,^63^ and are mainly covered by nurses. Conversely, the case manager, essential for coordinating integrated healthcare for frail individuals,^64,65^ was present in fewer than half of the facilities.

As advocated by the Global Action Plan 2017–2025, organizing and developing LTC systems for dementia requires providing care across the continuum from diagnosis to end-of-life, coordinating services among providers and system levels through multidisciplinary collaboration.^66^ Nevertheless, we found regional disparities in the integration of LTC networks, with Northern NHs better connected to territorial services such as day care centers and palliative care, and Southern facilities relying more heavily on rehabilitation.

Computerized medical records, known to improve care provision, health outcomes and reduce costs,^67^ were used in two-thirds of facilities, a proportion higher than that reported in a previous US study.^68^ However, adoption was significantly lower in the South compared with the North and the Centre. The use of fall-monitoring registers was generally high across macro-areas, with greater adoption in the North. Prior Italian evidence indicates that falls are more frequent in NHs than in other LTC settings, such as rehabilitation services.^69^ These findings underscore the need to standardize fall-reporting systems and the assessment of dementia-related risk factors, ranging from neurodegeneration and postural or gait impairments to psychotropic medication use and environmental hazards, to prevent severe outcomes such as fractures and mortality.^70^

In most NHs, people with dementia received an initial multidimensional assessment and regular follow-ups using widely adopted tools such as the Barthel ADL,^71^ MMSE,^29^ and the Braden^30^ or Norton scales.^72^ Although no studies have yet identified the most effective instruments for predicting key outcomes in NHs—such as mortality or hospital admission—the value of multidimensional assessment for residents in long-term care facilities is well recognized.^73^

Our findings indicate that the Northern and the Central NHs operated with a similar number of beds per professional, a ratio higher than that observed in Southern facilities. This suggests that many NHs may be operating under conditions that do not ensure safe and high-quality care, given the well-established association between staffing levels and resident outcomes, such as falls, pressure ulcers, and hospital admissions.^74^ However, we acknowledge that staffing numbers alone are not sufficient to ensure high-quality care and that other factors, such as the possession of appropriate competencies, also play a crucial role. Indeed, Northern and Southern facilities reported more training hours than those in the Central. Staff training is associated with reductions in residents’ BPSD and enhances staff self-efficacy and sense of competence.^75^

### Care services and treatments referred to 2019

The geographical differences also concern the provision of services, such as speech therapy, personal care services, and social and recreational activities, which were most widespread in Northern facilities. Therefore, it appears that facilities located in the North focused more on healthcare and assistive needs, likely reflecting the presence of residents with higher medical and functional requirements.^61^ In contrast, facilities in the South and Islands tended to provide more specialized medical care and psycho-social services, such as psychological support, social assistance, and occupational therapy, possibly due to a more heterogeneous resident population.^61^

Psycho-social and rehabilitation treatments are considered the first-line approach for managing BPSD.^76^ We found that occupational activities and cognitive stimulation were commonly provided in most Italian NHs. Facilities located in the South more often implement reality orientation therapy,^32^ reminiscence therapy,^33^ cognitive-behavioral therapy,^34^ sensory gardens,^35^ and dance-movement therapy,^36^ while those in the North tended to provide doll therapy,^37^ pet therapy,^38^ touch therapy,^34^ and Snoezelen^39^ more frequently. These regional variations may reflect differences in staff composition, such as the presence of psychologists within multidisciplinary teams.^77^

In such an uneven landscape, the Italian NHs appear far from reflecting the person-centered care model advocated in the healthcare settings.^17^

### Strengths and limitations

Our findings represent the first in-depth investigation of NHs hosting PWD across the national territory, which is crucial given the rapid increase in LTC demand. However, the 46% response rate calls for caution in interpreting results.

Moreover, a key limitation lies in the characteristics of the facilities that fully completed the survey. As they were predominantly private NHs operating under agreement, more frequently classified as R2D level, and tended to have a larger bed capacity, a selection bias could have been introduced, potentially skewing the results toward a more favorable depiction of the national landscape than the reality.

Nonetheless, the strength of our findings lies in the highly detailed data collected and its macro-areas-based analysis, revealing important disparities in the appropriateness and accessibility of NHs for PWD. This provides a foundation for more equitable and effective allocation of public health resources in LTC.

### Conclusions

Results from the first Italian survey on structural, organizational, and care characteristics of NHs for PWD reveal an uneven distribution of facilities and their features nationwide, reflecting challenges in appropriateness, accessibility, and affordability of NHs where PWD live.

First, ensuring appropriateness requires establishing national standards for NHs and their staff, defining indicators to assess care quality, and implementing health policies that promote person-centered practices by investing in staff training and suitable physical environments. Second, improving accessibility entails reducing territorial disparities and adapting facilities to environments capable of providing adequate, person-focused support. Finally, guaranteeing the affordability requires not only reducing the financial burden on families and increasing public funding, but also expanding flexible residential alternatives, such as homelike units and dementia villages, and supporting caregivers throughout the entire care trajectory, through strengthening home-based care services, in line with current European practice.

These represent a system-level challenge that demands a recognition of dementia as a complex healthcare condition, a strategic vision of the residential services, and coordinated planning by policy-makers.

## Supplemental Material

sj-docx-1-alz-10.1177_13872877261442226 - Supplemental material for A snapshot of Italian nursing homes for people with dementia: A national survey of 1671 facilities Supplemental material, sj-docx-1-alz-10.1177_13872877261442226 for A snapshot of Italian nursing homes for people with dementia: A national survey of 1671 facilities by Roberta Vaccaro, Patrizia Lorenzini, Francesco Giaquinto, Fabio Matascioli, Emanuela Salvi, Giulia Carnevale, Nicoletta Locuratolo, Nicola Vanacore, Ilaria Bacigalupo, and in Journal of Alzheimer's Disease
