# Supplementary material for: A snapshot of Italian nursing homes for people with dementia: A national survey of 1671 facilities
Source: J Alzheimers Dis. 2026 May 6;111(3):1335–49. doi: 10.1177/13872877261442226 (PMC13219793; doi:10.1177/13872877261442226)
Supplement: sj-docx-1-alz-10.1177_13872877261442226 - Supplemental material for A snapshot of Italian nursing homes for people with dementia: A national survey of 1671 facilities [file sj-docx-1-alz-10.1177_13872877261442226.docx]

**Supplemental Material**

**A snapshot of Italian nursing homes for people with dementia: A national survey of 1671 facilities**

**Supplemental Table 1**. Distribution of nursing homes (NHs) participating in the survey by Italian regions and by geographical macro-areas and ratio of estimated cases of dementia to available facilities. Data are expressed as N (%) or percentages (%).

| **Region or Autonomous Province (A.P.)** | **Total number of NH** | **NH participating in the survey hosting PWD** | | **NH that fulfilled**  **the data collection form**  **referred to 2019** | | **Estimated cases of dementia*** | **Ratio cases/NH** |
| --- | --- | --- | --- | --- | --- | --- | --- |
| Emilia Romagna | 419 | 192 | 45.8% | 124 | 29.6% | 90250 | 215 |
| Friuli Venezia Giulia | 102 | 17 | 16.7% | 9 | 8.8% | 26121 | 256 |
| Liguria | 199 | 88 | 44,2% | 54 | 27.1% | 38202 | 192 |
| Lombardia | 694 | 442 | 63.7% | 298 | 42.9% | 180896 | 261 |
| P.A. Trento | 56 | 55 | 98.2% | 47 | 83.9% | 9878 | 176 |
| P.A. Bolzano | 70 | 0 | 0.0% | 0 | 0.0% | 8512 | 122 |
| Piemonte | 616 | 173 | 28.1% | 101 | 16.4% | 90271 | 147 |
| Val d'Aosta | 5 | 2 | 40.0% | 0 | 0.0% | 2375 | 475 |
| Veneto | 352 | 188 | 53.4% | 143 | 40.6% | 90234 | 256 |
| **North** | **2513** | **1157** | **46.0%** | **776** | **30.9%** | **536739** | **214** |
| Lazio | 127 | 77 | 60.6% | 68 | 53.5% | 101195 | 797 |
| Marche | 180 | 89 | 49.4% | 53 | 29.4% | 32458 | 180 |
| Toscana | 324 | 174 | 53.7% | 102 | 31.5% | 79646 | 246 |
| Umbria | 71 | 28 | 39.4% | 25 | 35.2% | 19420 | 274 |
| **Centre** | **702** | **368** | **52.4%** | **248** | **35.3%** | **232719** | **332** |
| Abruzzo | 19 | 7 | 36.8% | 5 | 26.3% | 25778 | 1357 |
| Basilicata | 17 | 12 | 70.6% | 2 | 11.8% | 10661 | 627 |
| Calabria | 55 | 6 | 10.9% | 5 | 9.1% | 32666 | 594 |
| Campania | 73 | 14 | 19.2% | 9 | 12.3% | 78551 | 1076 |
| Molise | 7 | 7 | 100.0% | 3 | 42.9% | 6445 | 921 |
| Puglia | 138 | 86 | 62.3% | 50 | 36.2% | 68451 | 496 |
| Sardegna | 18 | 7 | 38.9% | 5 | 27.8% | 30364 | 1687 |
| Sicilia | 65 | 7 | 10.8% | 6 | 9.2% | 80453 | 1238 |
| **South and Islands** | **392** | **146** | **37.2%** | **85** | **21.7%** | **333369** | **850** |
| **Total** | **3607** | **1671** | **46.3%** | **1109** | **30.7%** | **1102827** | **306** |

*The estimated cases of dementia in a specific Region were estimated by multiplying the European dementia prevalence stratified by age and gender^1^ for the number of over-65-year residents in each Italian Region in 2022, as provided by the Italian National Institute of Statistics (http://dati.istat.it/).

1. Bacigalupo I, Mayer F, Lacorte E, et al. A systematic review and meta-analysis on the prevalence of dementia in Europe: estimates from the highest-quality studies adopting the DSM IV diagnostic criteria. *J Alzheimers Dis* 2018; 66: 1471-1481.

**The Permanent Table of the National Dementia Plan Study Group**

Gennarina Arabia (Catanzaro), Alessandro Amorosi (Milano), Ilaria Bacigalupo (Roma), Anna Maria Bargagli (Roma), Luisa Bartorelli (Roma), Cristina Basso (Padova), Manuela Berardinelli (Roma), Maria Pompea Bernardi (Catanzaro), Caterina Bianchi (Roma), Lorenzo Blandi (Pavia), Federica Boschi (Bologna-Ravenna-Modena), Amalia Cecilia Bruni (Lamezia Terme (CZ)), Alessandra Caci (Aosta), Paolo Cafarra (Parma), Marco Canevelli (Roma), Andrea Capasso (Napoli), Susanna Cipollari (Macerata-Roma), Mariapia Cozzari (Giovinazzo (BA)), Alfonso Di Costanzo (Campobasso), Teresa Di Fiandra (Roma), Annalisa Di Palma (Napoli), Andrea Fabbo (Bologna-Modena), Federica Francescone (Roma), Carlo Gabelli (Padova), Sabina Gainotti (Roma), Francesca Galeotti (Roma), Giuseppe Gambina (Verano), Marina Gasparini (Roma), Maria Assunta Giannini (Roma), Micaela Gilli (Trento), Marcello Giordano (Palermo), Annarita Greco (Napoli), Antonio Guaita (Abbiategrasso (MI)), Fabio Izzicupo (Senigallia (AN)), Fiammetta Landoni (Roma), Elisa Lidonnici (Genova), Nicoletta Locuratolo (Roma), Giancarlo Logroscino (Bari-Tricase (LE)), Alessandra Lombardi (Trento), Gilda Losito (Roma), Francesca Lubian (Bolzano), Maria Cristina Lupinetti (Pescara), Sara Madrigali (Firenze), Camillo Marra (Roma), Filippo Masera (Ancona), Massimiliano Massaia (Torino), Antonio Mastromattei (Roma), Antonio Matera (Potenza), Manlio Matera (Sesto Fiorentino (FI)), Francesco Mazzoleni (Sondrio), Carla Melani (Bolzano), Serena Meloni (Cagliari), Elena Memeo (Bari), Marco Musso (Torino), Antonella Notarelli (Firenze), Marco Onofrj (Pescara), Ernesto Palummeri (Genova), Valeria Panetta (Potenza), Carlo Petrini (Roma), Tommaso Piccoli (Palermo), Alessandro Pirani (Ferrara), Stefano Piras (Cagliari), Gabriella Porro (Milano), Mario Possenti (Milano), Elena Rendina (Roma), Antonino Riolo (Trieste), Luciana Riva (Roma), Emanuela Salvi (Roma), Sara Santini (Pescara), Silvia Scalmana (Roma), Nando Scarpelli (Perugia), Piero Secreto (Torino), Monica Seganfreddo (Aosta), Stefano Sensi (Chieti-Pescara), Carla Severino (Campobasso), Patrizia Spadin (Milano), Patrizia Spallino (Torino), Anna Laura Spinelli (Spoleto), Andrea Stracciari (Bologna), Marco Trabucchi (Roma), Nicola Vanacore (Roma), Antonio Zaccardi (Trieste)

**The NHs Study Group**

Abasi Manijet (Minerbio), Abrigo Enzo (Pocapaglia, Rodello), Accica Alessandro (Sessano Del Molise), Acerbis Paola (Arosio), Achille Antonella (Cigognola), Achilli Danilo (Stradella), Acquaviva Carlotta (Novafeltria), Airoldi Milena (Gorla Minore), Albeik Ahmad (Diano Marina), Alberghina Massimiliano (Annicco), Alberghini Maltoni Rugiada (Sanremo), Albertoli Barbara (Marchirolo), Albini Bruno (Borbona), Alessandroni Francesco (Fano), Alessi Santo (Acquasanta Terme, Ascoli Piceno), Alfani Mario (Montemagno), Alì Nicola (Lanzo D'Intelvi, Pellio Intelvi), Allemano Paolo (Sanfront), Aloise Giuseppe (Trevignano Romano), Alongi Salvatore (Montese), Alquati Enrica (Borgarello), Altieri Caterina (Fontanellato), Alvarez Chaves Ana Del Rosario (Robecco D'Oglio), Amantia Marilena (Dresano), Amato Giuseppe (Alba), Ambrosini Ilenia (Mantova, Marmirolo), Ambrosini Lisa (Quattro Castella), Ameglio Valtero (Savona), Amico Roberto (Bobbio), Anastasio Luigi (Filadelfia), Anchisi Giovanni (Novara), Andina Angelo (Oleggio, Pogno), Andreucci Barbara (Castelnuovo Di Garfagnana), Anela Maria Giovanna (Modena), Anfossi Laura (Voghera), Angelini Alessio (Ronciglione), Angellotti Giuseppe (Castellammare Di Stabia), Angioli Fabiana (Fauglia), Antinarelli Simonetta (Spoleto), Antoniadis Antonios (Ancona), Apollonio Nicholas (Cecina), Arangio Giovanni (Leonforte), Arangio Giuseppe (Riccione), Arbuffi Maura (Alessandria), Arcangeli Annalisa Vera (Palazzolo Sull'Oglio), Archetti Lorenzo (Castrezzato), Arduino Marco (Valle Lomellina), Argenteri Angelo (Gambolò), Armanni Lilli (Bienno), Arrobbio Cristiana (Poirino), Artusi Attilio (Barzio, Introbio), Arvati Marco (Mantova), Arvia Giuseppe (Mori), Assadi Fakri (Besano), Assandri Roberto (Rossiglione, Mele), Asteggiano Giovanni (Alba), Astegiano Roberto (Cherasco), Astolfi Massimiliano (Osimo), Astolfi Sabrina (Torino), Audia Paolo (San Giovanni In Fiore), Augeri Giorgio (Vallecrosia), Avanzi Giovanni Luca (Rovigo, Papozze), Azzali Stefania (Parma), Azzalini Angela (Cordignano), Azzarà Giorgio (Valbondione), Baalbaki Khaula (Cremona), Bacci Edi (Civitella In Val Di Chiana), Baccio Maria (Leonforte), Badagliacca Francesco (Bari), Badiale Mauro (Merlara, Sossano), Baffoni Elisabetta (Bucine), Baiano Raffaele Nilo (San Giorgio Del Sannio), Balbo Viola (Garessio), Baldini Franco (Gambassi Terme), Balduzzi Valerio (Belgioioso), Balice Walter (Otranto), Balletta Michele (Bologna), Balzaretti Renato (Appiano Gentile), Balzarini Marco (Castiglione Chiavarese), Banci Cinzia (Pitigliano), Baratelli Giuseppe (Cesena), Barbabella Giuseppe (Francavilla D'Ete), Barbato Elisabetta (Castelfranco Veneto), Barberi Anna (Folgaria), Barberini Sara (Modena), Barberis Giorgio (Calizzano), Barbieri Cristina (Modena), Barbieri Luisa (San Lazzaro Di Savena), Barbolini Elisabetta (Modena), Barca Vanna (Gallarate), Barca Vanna (Gallarate), Bargetto Gabriele (Cengio), Barro Raffaella (Caorle), Bartoleschi Giuseppe (Montefiascone), Bartolini Carla (Prato), Bartolucci Emma (Roma), Bartolucci Silvia (Pieve Santo Stefano), Barzaghi Alberto (Padova), Basile Paolo (Santa Marinella), Bassani Maria (Porto Ceresio, Lavena Ponte Tresa), Bassi Ezio (Ostiglia), Bassilici Antonella (Firenze), Basso Antonio (Albenga), Bastoni Fiammetta (Genova), Batocchio Paolo (Trichiana), Battegazzore Cesare (Ponte Nizza), Battisti Antonietta (Cologna Veneta), Battisti Basilio (Rieti), Battocchio Paolo (Mel, Lentiai), Bazzani Daniela (Porretta Terme), Beatini Marco (Campo Ligure), Beccia Federico (Bobbio), Bedin Loredana (Zero Branco), Beghi Gianfranco Maria (Pavullo Nel Frignano), Begnini Marco Alessio (Savignano Sul Rubicone), Belforti Vittoriano (Fontanellato), Bellato Marilena (Cervia), Belli Serena (Bleggio Superiore), Bellini Benedetto (Bari), Bellino Pier Paolo (Valdieri), Bello Mauro (Asiago), Bellocchi Luigi (Rosora), Belloni Giovanni (Arena Po), Belluomo Maria (Centallo), Belotti Gloria (Bergamo), Beltarre Roberta (Bellinzago Novarese), Beltempo Tiziana (Monte San Vito), Benvenuti Gabriella (Carmignano), Benvenuti Loredana Sonja (Medicina), Beretta Silvia (Gromo), Bergamaschi Luca (Stia), Bergamo Pamela (Livorno Ferraris), Bergianti Annarita (Montecchio Emilia), Berlengiero Claudio (Ventimiglia), Berlusconi Francesco (Sumirago), Bernardi Alberto (Pievepelago), Bernardi Daniela (Massignano), Bernardi Dario (Bassano Del Grappa), Bernardi Stefano (Gaggio Montano), Bertazzoni Daniela (Vimercate), Bertinato Giulio (Alano Di Piave), Bertoglio Piera (Romanengo), Bertola Diego (Dronero), Bertoli Giulia (Gussago), Bertolino Ilario (Boves), Bertuletti Giuseppe (Almenno San Salvatore), Bettini Alessia (Chiusdino), Bettini Fausto (Marcaria), Bettini Laura (Roe' Volciano), Bezhani Usela (Borgo Valsugana), Bezzi Achille (Fusignano), Bezzi Mario (Pontremoli), Biallo Martino (Adelfia), Bianchi Andrea (Loreto), Bianchi Marco (Uggiate-Trevano), Bianchi Marco (Viggiù), Bianchi Sabrina (Livorno), Bianchini Cinzia (Bomarzo), Bianciardi Andrea (Impruneta), Bianco Andrea (Caselle Torinese), Bianco Roberta (Rodello), Biason Antonella (Luino), Biffi Antonella (Erba), Bigazzi Anna (San Giovanni Valdarno), Binatti Andrea (Novara), Bini Maurizio (Signa), Biografi Arianna (Verucchio), Biondi Francesco (Figline Valdarno), Biondo Radames (Silea), Birra Emanuela (Sesto San Giovanni), Biselli Ermanno (Massa), Boca Barbara (Novara), Boccadamo Alberto (Corigliano D'Otranto, Taviano), Boccafogli Massimo (Vigarano Mainarda), Bocchi Cristiana (Piacenza), Bocchini Maria Federica (Ostra Vetere), Boemio Pasquale (Sant'Anastasia), Boggio Elisabetta (Prarostino), Boito Arrigo (Limana), Bold Mariana (Borgonovo Val Tidone), Boldini Annalisa (Piadena, Acquanegra Sul Chiese), Boledi Paolo (Rivergaro), Bolis Lidia Paola (Calcinaia), Bollati Paola (Marene), Bolognani Bruno (Mezzocorona, Mezzolombardo, Fondo), Bolognesi Mauro (Bergamo), Bombaglio Arnaldo (Dubino), Bonacina Carlo Ambrogio (Civate), Bonan Riccardo (Marostica), Bonatto Revello Sandra (Locana, Rivarolo Canavese), Bonaventura Domizio (Nago-Torbole), Bonello Franco (Borgomaro), Bonetti Manuela (Capannori), Bonferoni Piero (Somma Lombardo), Bonghi Michele (Besana In Brianza, Biassono), Bonini Maria Grazia (Busseto), Bonizzato Stefano (Marcaria), Bonollo Tullio (Saccolongo), Bonvini Flavio (Pianello Del Lario), Bordignon Neli Salete (Fratta Polesine), Borellini Elide (Berbenno Di Valtellina), Borghi Sabrina (Solbiate), Borroni Daniele (Vanzago), Borroni Giuseppe (Genova), Borsati Charitas (Mezzane Di Sotto, Bortot Giulia (Pieve Di Cadore), Boscaro Federica (Montagnana, Cologna Veneta), Boschetti Vittorio (Lendinara), Boscia Filippo (Alberobello), Bosetti Stefano (Predazzo), Bosio Stefano (San Germano Chisone), Botrugno Paolo (Cavriana), Botteri Francesco (Pianello Val Tidone), Bottoni Monica (Stienta), Bovello Daniela (Montaldo Scarampi), Boychenko Olena (Maranello), Braghenti Orazio (Vigo Di Fassa), Branchini Giovanni (Rivarolo Mantovano), Brandi Sivia (Carasco), Brandolani Lorenzo (Levico Terme), Brasini Luca (Cesena), Brau Maria Franca (Sassari), Brezzo Sara (Pocapaglia), Bricco Tiziana (Carcare), Brighi Dania (Cesena), Brunello Simone (Bovolone), Bruni Cristina (Castel Del Piano), Brunini Alessandro (Cecina), Bruno Antonio (Fiumicino), Bruno Fulvio (Schivenoglia), Bruno Irene (Bologna), Bruno Rosaria (Favria), Bruschi Bruno (Borgofranco Sul Po), Bruschi Debora (Pian Di Sco), Brustia Piero (Novara, Prarolo), Bruzzone Irene (Campomorone), Bubola Marta (Nogara), Bucalossi Chiara (Cascina), Buccarella Valentina (Sogliano Al Rubicone), Bucci Adelmo (Genova), Bucci Emma (Faenza), Buccieri Nicandro (Isernia), Bugada Giuseppe (Pontoglio), Bugatti Paola (Conzano, Moncalvo), Buggio Raffaella (Selvazzano Dentro), Bulgari Francesca (Legnano), Bulleri Mariella (Empoli), Bulzamini Paola (Bologna), Buoncristiani Silvia (San Giuliano Terme), Buongiovanni Carlo (Trento), Buonocore Serena (Cogoleto, Genova), Busnelli Roberto (Concorezzo), Buso Miriam (Venezia), Cabras Roberto (Rovato), Cacciabue Franca (Nizza Monferrato), Cacciatore Sergio (Gagliano Del Capo), Cacciatori Stefano (Trevenzuolo, San Giovanni Lupatoto, Minerbe, San Giovanni Lupatoto, Bosco Chiesanuova, Verona), Cafariello Carmine (Viterbo), Cagnolati Veronica (Parma), Caimi Barbara (Merate), Calabrese Giusi Alessandro (Terni), Calamari Carlo Antonio (Suzzara), Cambria Stefano (Gavirate, Somma Lombardo), Camilleri Fabio (Roma), Caminonni Romina (Cupra Marittima), Campanello Francesco (Pieve Del Cairo, Retorbido), Campedelli Anna Maria (Cesenatico), Campetella Valentina (Matelica), Canafoglia Corrado (Senigallia), Canali Francesca (Pietrasanta), Candiano Siro (Candelo), Canevari Bruna (Mantova), Cangenua Marco (Osimo), Caniglia Sebastiano (Villa Di Tirano), Canil Stefania (Cavagnolo), Cannone Alessia (Torino), Cantarella Alessandra (Mira), Cantore Martino (Barasso), Cantoro Antonio (Monopoli), Capelli Marco (Pianoro), Capoferri Federica (Dormelletto), Caponi Isabella (Montaione), Capotosto Emanuela (Treviso), Cappelli Caterina (Marliana), Capraro Rocco (Matino), Capretti Giovanni (Gargnano), Capuano Sofia (Santa Croce Sull'Arno), Caputo Giuseppe (Alezio), Capuzzi Donato (Castel Di Sangro), Carbonara Michele (Bitritto), Carbonaro Paolo (Sansepolcro), Carbone Gabriele Francesco Salvatore (Guidonia Montecelio), Carboni Francesca (Capoterra), Carbonieri Isabella (Maranello), Carlà Angelo (Legnano), Carlino Matteo (Riva Ligure, Sanremo), Carnuccio Anna Maria (San Mauro Pascoli), Carrano Francesco (Patrica), Carratelli Domenico (Roma), Carrozza Diego (Lizzano In Belvedere), Carrozzo Giovanni Pompeo (Mesagne), Cartocini Claudio (Vescovato), Cartosio Marco Sergio (Tonengo), Caruso Anna (Pellizzano), Caruso Valerio Giuseppe Maria (Santa Marinella), Casali Sofia (Vernasca), Casè Pierangela (Robbio), Casella Antonio (Milano), Casella Luigi (Ariccia), Caselli Erica (Modena), Caselli Massimo (Vignola), Caserta Maria (Fano), Cassani Ellena (Sumirago), Cassano Teresa (Ancona), Cassino Roberto (Pieve Del Cairo), Castagna Ilaria (Barbarano Vicentino), Castello Giuliana (Reggio Nell'Emilia), Castelnovo Cristiano Giovanni (Rosolina), Castiglioni Franco (Lazzate), Catalano Andrea (Villa Di Tirano), Catalano Ravaglioli Marta (Gaglianico), Cataldi Antonella (Bari, Casamassima), Cattaneo Daniele (Lainate), Cattaneo Monica (Mesero), Cavaglià Tania (Castelnuovo Don Bosco), Cavagnaro Mario (San Venanzo), Cavagnino Augusto (Orta San Giulio, Suno), Cavallaro Alessia (Ispra), Cavallaro Ermelinda (Bazzano), Cavalli Rossana (Porto Valtravaglia), Cavicchi Fabio (Camugnano), Cazzolla Roberto (Stagno Lombardo), Cazzulo Pietro (Torriglia), Cebanu Maria (Bellagio), Ceccardi Marco Dante (Malalbergo), Cecchin Fabio (Rosà), Cecchino Ada (Trento), Cecconi Chiara (Seravezza), Cella Giampaolo (San Martino In Rio), Cencetti Alba (Riolo Terme), Cenci Federica (Roma), Cenci Giuliano (Urbino), Cerri Alberto (Villa D'Adda, Treviolo), Cervasio Immacolata (Pavullo Nel Frignano), Cervigni Laila (Tolentino), Cesaretti Myriam (Rezzato), Cesari Giorgio (Trento), Cesarini Simonetta (Perugia), Ceschi Luca (Cologna Veneta), Cesena Desiree (Boretto), Chermaneanu Mihai Catalin (Cesena), Cheso Giuseppe (Bassano Del Grappa), Chianni Emilia (Certaldo), Chiaramello Paolo (Cuneo), Chiarelli Amedeo (Fontecchio), Chiarelli Grazia (Baricella), Chicco Maurizia (Bagnolo Mella), Chieppa Lara (Pieve Torina), Chiesara Maria Angelica (Monza), Chiesi Angelo (Bagnolo In Piano), Chimenti Maurilio (Massa), Chitò Eugenia (Roncadelle), Chizzoni Mariacristina (Marcaria), Chorbikj Izabela (Corsico), Ciaceri Donatella (Asti), Ciancia Domenico (Corato), Cianciosi Paola (Genova), Ciatti Annalisa (Siena), Ciavattini Carlo (Silea), Cimmino Sonia (Bologna), Cinelli Cinzia (Arezzo), Ciobanu Andra Laura (Bra), Ciommei Anna Maria (Subbiano, Arezzo), Cioni Sara (Bologna), Cipani Maria Fausta (Parma), Cipriani Silvia (Cortona), Ciracì Nicola (Mesagne, Ostuni), Cirasino Domenica Liliana (Ostuni), Ciravegna Rossana (Cherasco, Narzole), Cirelli Mario (Nembro), Cirelli Roberta (Predore, Spirano, Dalmine, Seriate), Cirillo Enrico (Peveragno), Cirillo Valentina (San Mauro Torinese), Cirinei Eleonora (Gubbio), Clerico Attilio (Monesiglio), Cognetta Francesco (Asso), Cogo Fabrizio (Pianezza), Coin Teresa (Padova), Colacicco Giuseppe (Monopoli), Colella Giuseppe (Fano), Collacchioni Vieri (San Godenzo), Collinelli Marco (Sarsina), Colloca Ermanno (Cesano Maderno, Limbiate), Colombetti Roberta (Crema), Colombo Cristina (Barlassina), Colombo Davide (Stra, Noventa Padovana, Orgiano), Colombo Gabriella (Dogliani), Colombo Luigi (Albavilla), Colombo Stefano (Sassoferrato), Colosio Pier Lorenzo (Storo, Pieve Di Bono), Colturi Gennj (Sondalo), Comerro Teresio (Front), Committeri Pier Vincenzo (Spello), Compiano Michela (Sestri Levante), Conconi Elisa (Casina), Confalonieri Donatella (Castiglione Delle Stiviere), Conforti Francesco (Pescia), Consani Alberto (Capannori), Consorzio Solco (Ravenna), Contessa Maria Antonietta (Bologna), Conti Monia (Faenza), Contro Paolo (Valstagna), Coppe Orazio (Treia), Coppedè Giovanna (Casciana Terme), Coppedè Sonia (Camaiore), Corcelli Francesco (Sangano), Corigliano Donatella (Roma), Corino Marco (Tromello, Sartirana Lomellina), Cornaglia Paola (Cerrina), Coronelli Maurizio Maria (Borgo San Siro), Corradini Anna (Borgo Valsugana), Corradini Lucio (Scandiano), Corsi Sergio (Amandola), Corsini Patrizia (Porretta Terme), Corso Massimo (Lucca), Corti Giulio (Premana), Corvaglia Anna Giuseppina (Roma), Coser Patrick (Trento), Cosimi Maria Laura (Montegiorgio), Costa Daniele (Curtatone), Costagliola Carmela (Napoli), Costantini Claudio (Calice Ligure, Collegno), Costantino Gianfranco (Manfredonia), Cottica Clarissa (Ficarolo), Cottinelli Maria (Lovere), Covili Paola (Castelfranco Emilia), Cozzi Roberto (Milano), Cresci Roberto (Milano), Crespi Pierernesto (Suno), Cresta Paola (Albisola Superiore), Criasi Antonio (Villafranca Piemonte, Virle Piemonte), Crispino Giorgio (Bocchigliero), Cristiano Ernesto (Montesilvano), Cristiglio Paola (Spotorno), Croce Alessandro (Verrua Savoia), Croce Elisabetta (Fabbrico), Croce Piermario (Moncrivello), Croce Bermondi Giuseppe (Campomorone), Crociani Stefano (Monteveglio), Croppi Gustavo (Ticineto), Crotti Barbara (Rodengo-Saiano), Crucinio Nicola (Alberona, Volturino), Cucca Nicola (Casalecchio Di Reno), Cuccato Andrea (Correggio), Cucchio Simona (Albese Con Cassano), Cuoghi Katia (Castelvetro Di Modena), Curcio Maddalena (Naro), Curti Carlo (Viterbo), Da Re Silvia (Tarzo), Daccico Sergio (Morciano Di Romagna), D'Acunto Alessandro (Trebaseleghe), D'Addio Annalisa (San Lorenzo In Campo), D'Agostino Vladimiro (Prato), Dal Ben Daniele (Portogruaro), Dal Molin Anna (Valdastico), Dal Pra Sara (Camisano Vicentino), D'Alba Maria Ada (Otranto), Dalessandro Giuseppe (Triggiano), Dalla Pozza Maddalena (Camisano Vicentino), Dalla Riva Cristina (Valdagno), Dalla Riva Sara (Soave), Dalla Vecchia Loretta (Sant'Anna D'Alfaedo), Dallera Cristina (Voghera), Dama Michele (Mongrando), D'Ambrosio Eleuterio (Fontecchio), D'Andrea Aldo (Volturara Irpina), D'Andrea Maurizio Silvano (Trasacco), D'Angelo Francesco (Borgo Val Di Taro), Danilovic Biljana (Guanzate, Mozzate), D'Anna Sebastiano (Eraclea), D'Antino Arianna (Monticello Brianza), D'Anza Elisa (Valfurva), D'Apote Michele (San Giorgio Di Piano), Dashtipour Mazeyar (Arco), D'Aversa Gianvito (Ostuni), Davoli Andrea (Manziana), Dazzani Stefania (Medicina, Imola), De Angelis Stefano (Urbisaglia), De Battisti Paon (Verona), De Clementi Francesca (Fonte Nuova), De Falco Gabriella (Ronco Briantino), De Faveri Piero (Conegliano), De Feo Riccardo (Cassano Magnago), De Gesù Roberto (Soliera), De Giambattista Luca (Valfurva), De Lorenzo Saverio (Grosio), De Luca Silvio (Covo), De Marinis Flavio (Sannicandro Di Bari), De Martinis Monica (Bedizzole), De Micheli Claudio Massimo (Bra), De Palo Francesco (Colleferro), De Patre Paolo (Arosio, Mariano Comense), De Pieri Maurizio (Treviso), De Pierro Giovanna (Giaveno), De Riva Gabriella (Fonzaso), De Rizzo Silvia (Trissino, Recoaro Terme), De Tullio Renato (Valenzano), Deambrogio Matteo (Biella), Debbi Jacopo (Casalgrande), Debenedetti Federica (Loano), Dedalo Claudio (Castelforte), Defrancisco Barbara (Alpignano, Torino), Degiovanni Daniela (Casale Monferrato), Degl’Innocenti Loretta (Camaiore), Deidda Emanuele (La Spezia, Levanto), Del Barba Sonia (Morbegno), Del Mistro Francesco (Grosotto), Del Priore Marina (Pareto), D'Elia Carlo (Venezia), Della Valle Maria Pia (Scarnafigi), Dellacasa Maria Francesca (Bogliasco), Dellarole Giuliana (Collegno), Delli Falconi Marta (Roma), Demaria Pietro (Stroppo), D'Errico Filomena (Cardito), D'Errico Rosanna (Copertino), Destro Tamara (Palestro, Novara), Dettoni Elisabetta (Castiglione Tinella), Devasini Manuela (Cinisello Balsamo), Di Bello Antonio (Monopoli), Di Bello Gaetano (Lauria), Di Buò Mariangela (Offida), Di Castri Massimo (Mesagne), Di Fabio Manuela (Parma), Di Fazio Giorgio (Cori), Di Folco Francesco (Roma), Di Giambattista Carlo (Chieri), Di Gioia Antonella (Bovolenta), Di Lenardo Fabio (Codroipo), Di Lorenzo Giuseppe (Oria), Di Maggio Maria Grazia (Oggiono), Di Matteo Antonio (Agliè), Di Matteo Franco (Rocca De' Baldi), Di Nardo Angela (Premosello-Chiovenda), Di Pietro Luigi Giuseppe (Trieste), Di Stefano Ivano (Roma), Dimori Sergio (Vedano Olona, Sesto Calende), D'Innocenzo Silvia (Livorno), Dionisi Angelo (Cittaducale), Dipasquale Giuliano (Coccaglio, Seniga), Diurisi Giovanni (Soleto), Divulsi Deborah (San Michele Mondovì), Dogaru Ionut Daniel (Trieste), Dolcini Franco (Castelfidardo), Domenico Romano (San Lazzaro Di Savena), Donà Francesco (Venezia), Donadio Paola (Firenze), Donatella Vianello (Venezia), Dorigoni Nicoletta (Baselga Di Pinè), Dotta Federica (Luserna San Giovanni), Drioli Stefano (Tarzo), Durini Ernesto (Alezio), D'Urso Leucio Antonio (Cene), Dyrma Blerina (Carpenedolo), Egidi Isabella (San Benedetto Del Tronto), El Majdoub Meryem (San Benedetto Po), Empiri Silvio (Borgomanero), Enea Anchise (Pergola), Enrico Gianluca (Andorno Micca), Epis Renzo (Tirano), Errico Serena (Molinella), Errico Serena (Galliera), Espen Kati (Baselga Di Pinè), Esposito Anna (Cameri), Esposito Eduardo (Borgo San Lorenzo), Esposto Elisabetta (Mombaroccio, Pesaro), Fabbiano Sabrina (Torino), Fabbrini Stefano (Vimodrone), Fabietti Elisabetta (Figline Valdarno), Fabio Leo (Sasso Marconi), Fabio Zucca (Casnigo), Facci Francesco (Este), Falappi Monica (Firenze), Falletti Giovanni (Front), Falone Luca (Torino), Falso Gianfranco (Minturno), Fambrini Alessandra (Camaiore), Fancelli Silvia (Cecina), Fantozzi Massimo (Pescia), Fanucci Claudia (Pisa), Fariello Francesco (San Miniato), Farina Piermario (Belgioioso), Fasano Giambattista (Santeramo In Colle), Fascetti Valeria (Genova), Fasciolo Piergiorgio (Genova), Fatone Daniele (Firenze), Favelli Felicita (Arezzo), Faverzani Mario (Ponte Di Legno), Favro Vito (Avigliana), Fazzalari Elisabetta (Perloz), Feder Francesca (Oppeano), Fedi Virginia (Pandino), Fellini Giuliano (Taviano), Feroldi Zaira (Rivoli), Ferrara Vito (Cassano Delle Murge), Ferrari Angela (Pellegrino Parmense), Ferrari Chiara (Casciago), Ferrari Cinzia (Santhià), Ferrari Fulvio (Sanremo), Ferrari Giuseppe (Varese), Ferrari Marco (Roma), Ferrari Massimo (Sorbolo, Senna Lodigiana), Ferrario Guido Luca Matteo (Milano), Ferrazza Andrea (Lonato), Ferrentino Aurelio (Pellegrino Parmense), Ferrero Elena (Monesiglio), Ferretto Maurizio (Arosio), Ferri Stefano (Valeggio Sul Mincio), Ferro Giuseppe (Ronco All'Adige), Ferro Rosalia Maria (Mombaroccio), Ferro Sara (Galliera), Fiandri Paola (Modena), Fiaschi Andrea (San Marcello Piteglio), Fiaschi Silvia (Certaldo), Fini Massimo (Sabaudia), Fino Gianluca (Beinette), Fioravanti Antonio (Fano), Fiore Francesca (Gaiole In Chianti), Fiore Giovanni (Mesagne), Fiorella Giuseppina (Aulla), Fiori Anna Vittoria (Oderzo), Fiorini Antonia (Artognem, Darfo Boario Terme), Fiscella Gianfranco (Sanremo), Florian Andrea (Torino), Florian Gabriella (Montebello Vicentino), Focone Nicoletta (Camerano), Foglia Gianmaria (Bari), Fogliata Moica (Solferino), Follin Francesco (Conegliano), Fomasi Laura (Morbegno), Fontana Giuseppe (Lavis), Fontana Ilaria (Godiasco), Fontana Karin (Castiglione Dei Pepoli), Fontanini Fulvio (Genova), Forcella Arnaldo (San Pietro In Casale), Forciniti Margherita (Prato), Forconi Stefania (Villanova Mondovì), Formica Valentina (Venaria Reale), Fornaciari Bruno (Masserano), Fornaro Simona (Edolo), Fornasari Tania (Reggio Nell'Emilia), Forni Roberto (Bologna), Forti Angelo (Castenedolo), Forti Fausto (Saronno), Foti Giovanna (Scilla), Frabetti Maurizio (Sant'Olcese, Genova), Franceschetto Giuseppe (Cavaso Del Tomba, Spresiano), Franceschina Emilio (Villafranca Padovana), Francesco Follin (Farra Di Soligo), Francesconi Andrea (Camaiore), Franchi Caterina (Siena), Franci Luisa (Sorano), Francia Rosanna (Forli'), Franco Alessandro (Montescudo, Rimini), Franco Bernardo (Fregona), Franzini Bruno Giovanni (Casalmorano), Frascisco Mauro Felice (Torino), Frate Fausto (Morbegno), Fratello Calogera (Lucca), Frea Bruno (Corneliano D'Alba), Freddo Francesco (Cupramontana), Frigerio Norma (Frascarolo), Frigieri Riccardo (Fiorano Modenese), Fumagalli Davide (Brivio), Funes Gianluca (Sedico), Furiosi Domenico (Lodi), Furlan Anna (Gorizia), Furlanetto Nilo (Pederobba, Furlani Gilberto (Boretto), Fuschini Aldo (Canzo), Gabaldo Fabrizio (Casale Di Scodosia), Gabrieli Efrem (Schilpario, Berzo Inferiore), Gaddi Marco (Torino), Gaetani Luigi (Arcinazzo Romano), Gaiardoni Carlo (Nogarole Rocca, Villa Bartolomea), Gaibisso Carlo (Pieve Di Teco), Galaschi Donatella (Zeme), Galasso Matteo (Sigillo), Galbiati Antonella (Cinisello Balsamo), Galeaz Francesca (Trento), Galeazzi Lucilla (Comano), Galizzi Pierangelo (Brescia), Gallo Cecilia (Chiavari, Rapallo), Gallo Cecilia Marisa (Favale Di Malvaro), Gallo Clemente (Scarnafigi), Gallo Franco (Genova), Gallo Stefano (Camposampiero), Gallon Dario (Casale Monferrato), Gallucci Augusto (Spiazzo), Galuzzi Cristina (Retorbido), Gamberini Maria (Modena), Garaffa Francesco Fabio (San Giorgio Canavese), Garavina Iader (Bellaria-Igea Marina), Garbella Paola (Occhieppo Inferiore), Garbin Stefano (Rossano Veneto), Garbolino Stefano (Cavour), Garrè Stefano (Montoggio), Garulli Michele (Neviano Degli Arduini), Gasparini Carlo (Vergato), Gasparri Damiano (Santa Marinella), Gasperi Diego (Certosa Di Pavia), Gastaldi Daniela (Milano), Gastaldi Margherita (Genova), Gatto Piergiorgio (Chiavari), Gattoni Chiara (Dormelletto), Gavelli Antonella (Bagnacavallo), Gaverini Mirko (Clusone), Gazzotti Alessia Maria Amore (Maranello), Genna Costanza (Siena), Gentile Luigina (Umbertide), Gentile Simona Alessandra Carmen (Cremona), Gerbasi Rosario (Racconigi), Gerli Diana (Vimercate), Gerola Roberta (Ispra), Ghetti Greta (Lugo), Ghiddi Francesca (Sassuolo), Ghielmetti Diego (Uggiate-Trevano), Ghilardi Ugo (Castelcovati), Ghinea Carmen Amelia (Mercato Saraceno), Ghio Rosita Giannina (Aulla), Ghiroldi Federica (Cecina), Ghisellini Michela (Castelmassa), Ghisetti Mariangela (Vailate), Ghiyasaldin Shahram (Canosa Di Puglia, Trinitapoli), Giacobbo Maria (Isola Vicentina), Giacomucci Pompilio (Casalbordino), Giacon Marco (Montebelluna), Giacopelli Guido (Segrate), Giacopini Claudia (Gambara, Quinzano D'Oglio), Gianatti Denis (Berbenno Di Valtellina), Gianazza Giulio Antonio (Cantello), Giannoni Federica (Castelnuovo Val Di Cecina), Giannotti Romano Mauro (Castelnuovo Di Garfagnana), Giardina Michele (Gravina Di Catania, Noicattaro), Giaretta Barbara (Rodigo), Gigli Giorgio (Castiglione Dei Pepoli), Gilardi Gabriele (Roccavione), Giordano Ettore (Vicoforte), Giordano Laura (Neive), Giordano Stefania (Roma), Giors Monica (Susa), Giossi Attilio (Porlezza), Giotti Chiara (Montevarchi), Giovannelli Giorgio (Roma), Giovannetti Gabriele (Barga), Giovannini Anna Valeria (Pinzolo), Giovetti Ferruccio (Sospiro, Isola Dovarese), Girelli Paolo (Goito), Giudici Guido (Clusone), Giuliani Nicola (Foggia), Giulisano Cecilia (Rho), Giusti Luigi (Pitigliano), Giusti Tamara (Palaia), Gobbetti Mara (Arcole), Gogna Luigi (Pralboino), Gori Maria Cristina (Terracina), Gotti Gian Marco (Siena), Grandi Debora (Gonzaga), Grandi Nicola (Padenghe Sul Garda, Botticino), Grandini Alessandro (Rivoli), Grassi Laura (Mergo), Grassi Vincenzo (Sarteano), Grassini Patrizia (Livorno), Graziani Manuela (Rimini), Greco Alessandro (Cles), Greco Costantino (Latiano,Ostuni), Greco Giovanni (Carmiano), Gregnanin Vanja (Giaveno), Gretter Andrea (Trento), Grigollo Barbara (Cameri), Grillo Antonio (Milano), Grizzetti Giovanna (Appiano Gentile), Grossi Luca (Stienta), Gualdi Irina (Capriate San Gervasio, Verdello, Cavenago Di Brianza), Gualdrini Alberto (Faenza), Guandalini Claudia (Modena), Guandalini Fausto (Comacchio), Guarcini Angela (Zagarolo), Guaricci Giuseppe Nadir (Altamura), Guarneri Bruno (Orzivecchi), Guarnieri Anna Maria (Castiglione Chiavarese), Guelfi Massimo (Fivizzano), Guerra Emiliano (Odolo, Sabbio Chiese), Guerrini Rocco Agnese (Chiari), Gugliotta Domenico (Turi), Gugliotti Giovanni (Isernia), Guidi Giovanni (Mondavio, Mondolfo), Guidi Pamela (Reggio Nell'Emilia), Guiso Gustavo (Selargius), Gurin Elisa (Sozzago), Guzzetti Lorenzo (Grosio), Iacomucci Marco (Pesaro), Iacovella Giuseppe (Castrocielo), Iafrate Piero (Ponsacco), Ianielli Leonardo (Rovereto), Iannaccone Vincenzo (Termoli), Iannielli Leonardo (Rovereto), Impiglia Nella (Serra De' Conti), Inguscio Cesare Giuseppe (Copertino), Insardà Michele (Gaggio Montano), Izzo Chiara (Roma), Jani Milla (Trieste), Jomiru Ala (Luzzara), Jovan Leci (Bologna), Kanyinda Tshilumbayi (Castellarano), Karakaci Fabiola (Brisighella), Kasapi Evangelia (Bologna), La Greca Davide Angelo (Nova Milanese), Labati Giuseppe (Rivergaro), Lachi Alberto (Porto Valtravaglia), Lamberti Francesco (Poggio Rusco), Lana Mauro (Borgoforte), Lancellotti Francesca (Bazzano, Bologna), Landra Pietro (Torino), Lanzeni Felice (Bergamo), Laperni Marco (Cazzano Di Tramigna), Larizza Giovanni (Altamura), Latella Vitaliano (Volpiano), Latini Fiorenza (Fanano), Latino Guido Salvatore (San Marco D'Alunzio), Lattante Loredana (Marino), Laurino Pasquale (Taio), Lavagetto Giampaolo (Traversetolo), Lavatori Paolo (Ripe), Leasi Eric (Compiano), Leci Jovan (Sasso Marconi), Leli Pietro (Borgo San Dalmazzo, Saluzzo), Lenti Ciro (Ostiglia), Leo Raffaella (Cassina De' Pecchi), Leonardi Lara (Rosa'), Leone Carlo (Apricale), Leone Flora Maria (San Mauro Torinese), Leone Francesco (Bollate), Leone Giuseppe (Gattinara), Leone Marcello (Giovinazzo), Lestingi Luigi (Adelfia), Letta Maria Luisa (Serra San Quirico), Levorato Marco (Verona), Li Bergolis Lucia (Monza), Licini Paola (Pieve Di Soligo), Liebschner Sabina (Paderno Dugnano), Linda Leoni (Correggio), Liveri Alessandra (Sant'Agata Bolognese), Lo Monte Francesca (Galbiate), Loaiza Mirian (Cerrina), Loda Giuliano (Capriano Del Colle, Brescia), Lodi Lorenzo (Bezzecca), Lombardi Alessandro (Castelfiorentino), Lombino Michele (Vittuone), Lomonaco Marco (Anzio), Lomurno Giuseppe (Assisi), Longo Cosimo (Trepuzzi), Longo Maria Grazia (San Giovanni Rotondo), Lorenzetti Barbara (Gazzo Veronese), Lorenzini Cinzia (Bibbona), Losi Vittorio (Modena), Lovi Emiliano (Lucca), Lozzi Andrea (Codogno), Lucca Nadia (Pisogne), Lucchesi Rosanna (Borgo A Mozzano), Luchetti Claudio (Novafeltria), Lulleri Sabrina (Siena), Lunardi Renato (Monselice), Lupi Giampietro (Paullo), Lupini Gabriele (Roma), Lussignoli Giulia (Brescia), Lutti Alberto (Polinago), Macchi Maurizio (Cerro Maggiore, Bodio Lomnago), Maccianti Enrico (Lonate Pozzolo), Madami Lallo (Corato), Madoglio Graziano Giulio (Chiomonte), Madridali Marta (Buti), Madrigali Marta (Pontedera, Lucca), Maffeis Cesare (Villa D'Alme', Casazza), Maglietta Rocco Alessandro Giuseppe (Potenza), Magliocchetti Caterina (Panicale,Citta' Della Pieve), Magnavacchi Mimma (Albinea), Magro Roberta (Trieste), Maiorana Elisa (Roma), Malacrida Patrizia (Castel San Niccolo'), Malatesti Cinzia (Montespertoli), Malavasi Luca (Bologna), Maltese Monica (Milano), Mammola Aldo (Villanova Mondovi'), Manara Giovanna (Brentonico), Manassero Alberto (Villafalletto), Mancinella Angelo (Roma), Mancini Antonia (Chignolo Po), Mancini Antonino (Isernia), Mancini Barbara (Rapolano Terme), Mancini Maria Luisa (Perugia, Marsciano), Mancuso Daniela (Lessona), Mandich Alessandra (Merone), Manfredi Laura (Ficarolo), Manfredi Sabato (Trezzo Sull'Adda), Manganelli Francesco (Castellanza), Mangano Cettina (Tesero), Mangano Mariangela (Moncalieri), Mango Francesco (Bovino), Mannino Beatrice (Parma), Mannino Salvatore (Alzano Lombardo), Mannozzi Alice (Firenze), Mannucci Simona (Montelupo Fiorentino), Manoni Claudia (Ostra), Manotti Andrea (Parma), Manto Giuseppe (San Canzian D'Isonzo), Mantovani Mauro (Ficarolo), Manzi Carla (Campiglia Marittima), Manzoni Paola (Merate), Marangon Federica (Meolo), Marangon Federica (Galliera Veneta), Marangoni Laura (Saltara), Marasca Antonio (Roma), Marasco Rolando (Carpi), Marazita Rosetta (Sarsina), Marazza Michele (Poli), Marcacci Maria Grazia (Citta' Della Pieve), Marcati Prisca (Vallarsa), Marcelli Claudio (Pieve Santo Stefano), Marchese Gianfranco (Firenze), Marchesini Giulio (Zevio), Marchetti Piera (Aprilia), Marchetto Roberta (Treviso), Marchi Spartaco (Faenza), Marchica Concetta (Misterbianco), Marconi Emanuela (Treviolo), Marconi Massimo (Ascoli Piceno), Marcucci Elisa (Todi), Marenzi Anna Maria (Palosco), Maretto Barbara (Legnano), Margheri Sandra (San Giorgio Di Piano), Marianetti Massimo (Genzano Di Roma), Mariani Emanuela (Caorso), Mariani Lorena (Borgo A Mozzano), Mariani Mirella (Desio), Marin Silvana Giovanna (Osio Sotto), Marini Alessandro (Corinaldo, Arcevia), Marini Marina (Monteriggioni), Marino Annunziatella (Senago), Marino Filippo (Aprilia), Marino Sebastiano (Chiuro, Floridia), Mariotti Isabella (Malonno), Maroni Paola (Ancona), Marrè Brunenghi Alessandra (Casalbuttano Ed Uniti), Marsico Pio Antonio (Monte San Savino), Marsili Alfredo (Montalto Delle Marche), Marsili Renzo (Porto Tolle), Martellosio Viviana (Vigevano), Martignano Roberto (Parabita), Martina Erika (Caselle Torinese), Martinazzoli Carlo Eugenio (Bollate), Martinazzoli Maria Luisa (Capo Di Ponte), Martinelli Arianna (Cento), Martinelli Gabriele (Terni), Martinelli Sandro (Borgoforte), Martini Camilla (Sanremo), Martini Dalila (Poggio A Caiano), Martini Di Cigala Elisa (Monticiano), Martino Francesco (Torino), Martino Gennaro (Faleria), Martinoli Carla (Breme), Masala Renato (Latina), Mascolo Massimo Domenico (Grado), Masini Simona (San Casciano In Val Di Pesa), Mason Silvana (Noale), Masperi Patrizia (Argenta), Massari Giancarlo (Asola), Massari Luca Maria (Magenta), Massari Olga (Guastalla), Massaro Clara (Milano), Massaro Clara (Milano), Massarotti Milena (Rovasenda), Massimo Alessi (Cartigliano), Massoli Stefano (Collazzone), Massoni Augusta (Rivanazzano), Mastroianni Gaetano (Milano), Matarazzi Cristian (Gualdo Tadino), Mattarelli Mauro (Bologna), Mattei Francesca (Bologna), Matteoni Samantha (Podenzana), Mattivi Maria Teresa (Pergine Valsugana), Mayan Sussele (Perledo), Mayan Sussele (Valmadrera, Lecco, Talamona), Mazzanti Mara (San Lazzaro Di Savena), Mazzarella Giuseppe (Sant'Elia Fiumerapido), Mazzetti Francesca (Sarteano), Mazzi Paolo (Ronco All'Adige), Mazzini Massimo (Vigevano), Mazzocato Andrea (Scorzè), Mazzotti Gian Primo (Cotignola), Meazza Daniele Marcello (Briosco), Medea Alessandra (Varese), Medugno Alfonso (Bellizzi), Meduri Antonino (Delebio), Meggiolaro Gianmario (Caldiero), Melandri Barbara (Imola), Mele Paola (Milis), Menculini Giuseppe (Perugia, Magione, Città Della Pieve), Meneghello Francesca (Noale), Meneghin Michela (San Pietro Di Feletto), Menetti Lorenzo (Monterenzio), Menghini Fulvio (Gandino, Brembate Di Sopra), Mengoli Pietro (Botrugno), Mercandelli Anna (Savona), Merella Mariaelena (Puos D'Alpago), Merletti Sergio (Cannobio), Merli Giuseppe (Carpaneto Piacentino), Merola Nicola (Castel Del Giudice), Mesini Benedetta (Soliera), Mezzadra Chiara (Pinarolo Po), Mialtu Daniela (Maranello), Micci Valentina (San Lorenzo In Campo), Miccoli Giovanni (Oria), Miceli Loredana (Ravenna), Migliosi Giovanni (Toscolano-Maderno), Milan Graziella (Napoli), Milani Franco (Canneto Sull'Oglio), Milardi Laura (Alassio), Mingrone Rosa (Duino-Aurisina), Minuti Anna Lisa (Monza), Mion Raffaella (Venezia), Miotti Viviana (Cerro Maggiore), Miracca Alessandra (Vidigulfo), Miravalle Costantino (Cafasse, Lanzo Torinese, Torino), Misserini Francesco (Isorella, Calvisano, Volta Mantovana), Mocchetti Fabio (Busto Garolfo), Mocellin Roberta (Castel San Giovanni), Modina Enrico (Carmagnola, Santena), Mogianesi Bedosti Elisabetta (Bologna), Mohiddin Samir (Bovegno), Mola Ernesto (Galatina), Molteni Gianbattista (Alzate Brianza), Monaco Giuseppe (Napoli), Monari Giuliano (Sestola), Mondino Marco (Montemagno), Mondo Laura (Santo Stefano Belbo), Moneghini Edi (Bagolino), Moneta Simone (San Fermo Della Battaglia), Moneti Paolo (Bagno A Ripoli), Moni Antonio (Città Di Castello), Monnecchi Alessandra (Castellina In Chianti, Castelnuovo Berardenga), Montagnani Stefano (Argenta), Montalbano Maria Grazia (Bomarzo), Montali Martha (La Spezia), Montanari Luca (Lavagna, Rapallo), Montanaro Alfonso (Rimini), Montanaro Maria Pia (Castelnuovo Calcea), Montanaro Serena (Dosolo, Viadana), Montanera Pier Giorgio (Aosta), Montaruli Angelo (Montecosaro), Montefusco Cecilia (Perugia, Assisi), Montesi Isabella (Fano), Monti Chiara (Venezia, Quarto D'Altino, Villadose), Monti Patrizia (Civitanova Marche), Monticone Matteo (Piossasco), Monticone Silvia (Paderno Dugnano), Montinaro Anna Maria (Calimera), Monzini Milvia (Milano, Tradate, Cassano Magnago), Mora Romina (Poggibonsi, San Gimignano, Colle Di Val D'Elsa), Morabito Francesco (Gaglianico), Morandi Mario (Mantova), Moraschini Renzo (Pergola), Morelli Eleonora (Macerata), Morelli Elisa (Calcinaia), Morelli Ivano (Robbio), Moretto Cristian (Gropello Cairoli), Morgantini Antonello (Ancona), Morini Caterina (Casalgrande), Morini Elio (Illasi), Morra Guglielmo (Beinasco), Morsa Angela (Pozzuoli), Mortola Maria Grazia (Rapallo), Moscato Brunetta (Signa), Mosconi Raoul (Russi), Mosconi Stefania (Brescia), Mossa Giovanni (Cassano Delle Murge), Mosso Tiziana (Albino), Mostarda Alessandro (Perugia), Mozzo Eleonora (Nuvolera), Mugnai Dina (Comacchio), Muiesan Sergio (Cormons, Gorizia), Munari Diego (Brendola), Munteanu Doina (Carpi), Muratore Enrico (Varazze), Muratori Giordana (Correggio), Musolino Filippo (Pecetto Torinese), Musto Antonio (Trezzo Sull'Adda), Muti Ettore (Mantova, Marmirolo), Nannelli Sabrina (Reggello), Nannetti Anna Rosa (Bologna), Napoliello Generoso (Villanuova Sul Clisi), Nardelli Giuseppe (Ventimiglia, Pigna, Dolceacqua), Nardi Sabrina (Como, Lipomo), Narracci Ottavio (Racale), Nasuti Emilio (Fontecchio), Natali Alessandra (Torre Di Mosto), Negrini Silvana (Ardenno), Nesladek Nerio (Trieste), Nespeca Maria Teresa (Ripatransone, Montefiore Dell'Aso), Netti Nicola (Cercola), Niccolini Paola Simona (Isola Del Cantone, Busalla), Nicoletti Laura (Cervia), Nicolotti Vincenza (Montichiari), Nicolucci Peppino (Aprilia), Nicosia Vincenza (Pinerolo), Nizzoli Silvia (Reggio Nell'Emilia), Nodari Lino (Orzivecchi), Nolasco Angelo (Bari), Novarese Irene (Montechiaro D'Asti), Novelli Antonio (Loreto, Campofilone), Novi Riccardo (Castelfranco Di Sotto, Fauglia), Novkovic Goran (Mediglia, Besana In Brianza), Nunziata Francesco (San Nicola Arcella), Oberti Sara (Leffe), Obici Francesco (Cologna Veneta), Occhi Giuseppe (Bormio), Oddi Alberto (Davagna), Odone Maria Giovanna (Oviglio), Ogien Matan (Laveno-Mombello), Ogniben Flavio (Monastier Di Treviso), Oliva Carlo (Nemi), Olivero Riccardo (Carcare), Olivetti Giuseppe (Corinaldo), Olivieri Marina (Busto Arsizio), Olivieri Susanna (Lucca, Gallicano), Olivotto Livio (Santo Stefano Di Cadore), Olla Maria Teresa (Grottammare), Ongaro Alberto (Urgnano), Ongis Gian Antonio (Somaglia), Orbecchi Christian (Alpignano), Orlandi Enrico (Santa Maria Della Versa), Orsi Filippo (Loreto), Orsi Flavia (Mirandola), Orti Marzio (Albonese), Ortolan Dania (Ponte Di Piave), Ortolani Alessandra (Bologna), Ortolani Anna (Imola), Ottobri Barbara (Pezzaze), Pacchioni Maurizio (Trigolo), Pace Michele (Monopoli), Pacifici Giovanni (Tivoli), Padovan Maurizio (San Donà Di Piave), Padroni Rosalba (Civitavecchia), Padrono Lorenzo (Tolfa), Paganini Pierangelo (Rapallo), Pagliara Riccardo (Foggia), Paialunga Ivana (Ascoli Piceno), Paindelli Mariagrazia (Ponte In Valtellina), Paknegad Saneh Kamran (Roma), Paladino Irene (Roma), Palazzo Giuseppe (Copparo), Palei Sabrina (Cortona, Castiglion Fiorentino), Palma Lucia (Medicina), Palmieri Carla (Fabriano), Palmieri Sonia (Roccastrada), Palmisano Silvestro (Locorotondo), Paltrinieri Renzo (Millesimo, Cairo Montenotte), Panaia Rocco (Cumiana), Pancera Federica (Marcaria), Panebianco Salvatore (Castel Del Piano), Panella Daniela (Ariano Nel Polesine, Taglio Di Po, Corbola), Panella Lorenzo (Marcignago), Panella Pasquale (Ferrera Erbognone), Panero Dario (Fossano), Paniccià Lorella (Sant'Elpidio A Mare), Paniga Sonia (Morbegno), Panni Graziano (Ostra), Pannilunghi Valerio (Pieve Tesino, Grigno), Pansini Alfredo Dionisio (Canosa Di Puglia, Trani, Mottola), Pantaleoni Nicola (Treviso), Panzalis Manuela (Genova), Paolinelli Barbara (Jesi), Paolinelli Barbara (Staffolo, Apiro, Cingoli), Papageorgiou Costantino (Torre Boldone), Papagno Elvira (Lucca), Paragò Giuseppe (Gerenzano), Parini Francesca (Piancogno), Parmeggiani Alessandra (Montechiarugolo), Parnanzini Franco (Monastir), Pascale Lisangela (Bitonto), Pascucci Francesco (Fossombrone), Pasqualetto Michele (Arsiero, Breganze, Caltrano), Passamonti Francesca (Porto San Giorgio), Passarini Lorenzo (Vestone), Passaro Jessica (Sarnico), Passaseo Emanuele Gaetano (Scarlino), Passera Egidio (Verdello), Pastore Silvia (Legnago), Pastorelli Paola (Giussano), Pataleo Luana (Nociglia), Patap Mihaela (Verrua Savoia), Pavan Giorgio (Mogliano Veneto), Pavano Gaetano (Sala Comacina), Pazienza Francesco (Panni), Pazzaglia Barbara (Merone), Pedrazzoli Roberto (Gonzaga), Pedretti Luca (Sant'Agata Bolognese), Peirano Emanuele (Barge), Pelanconi Laura (Chiavenna), Peli Roberta (Mazzano), Pellegrino Domenico (Appiano Gentile), Pellicanò Emanuele (Firenze), Pelosi Maria Teresa (Manerbio), Penco Stefania (Santa Margherita Ligure), Pennesi Monica (Montecosaro), Penzo Piergiorgio (Chioggia), Perati Gianluigi (San Bassano), Peraudo Piero (Torino), Perin Daniela (Pianiga), Perozzo Maurizio (Bassano Del Grappa), Perteghella Daniele (Rivanazzano), Perticone Francesco (Petrizzi), Peruffo Marco (Schio), Perutto Pierpaolo (Venezia), Pesaran Seyed Ali Bonakdar (Rovereto), Pesce Giampaolo (Savona), Pesel Giuliano (Duino-Aurisina), Petitti Di Roreto Cristina (Induno Olona), Petriello Lara (Levanto), Petrò Emiliano (Soncino), Pezzoli Michela (Roma), Pezzoni Damiano (Capriolo), Piana Antonietta Isabella (Genova), Piana Gianna (Genova), Piana Marco (Ottobiano, Valle Lomellina), Piana Paola (Candia Lomellina), Piazza Paolo (Belluno), Piazzolla Federica (Milano, Malnate), Pica Domenico (Nizza Monferrato), Piccinini Lino (Comano), Piccoli Massimo (Pescantina), Piedicorcia Luisella (Godiasco), Pierini Mara (Recanati), Pierobon Cinzia (Ponte Nelle Alpi), Pietrolino Angelo (Parona), Pietropaolo Maria (Montecompatri), Pignata Maurizio (Grugliasco), Pilotti Cristina (Sedico), Pilumeli Emilia (Vaglia), Pinelli Ignazio (Ragusa), Pini Alessandro (Bagno A Ripoli), Pini Vania (Bagno A Ripoli), Pinocci Gaia (Gallicano), Pinto Matilde (Piombino), Piralla Alberta (Orta San Giulio), Pirlo Maria (Martinengo), Piroli Remo (Soragna), Piron Emanuela (Transacqua), Pirondi Gianluca (Sala Baganza), Pisani Antonio (Fiuggi), Piscopo Andrea (Scisciano), Pistillo Mirella (Castel Goffredo), Piva Raffaella (San Giovanni In Marignano), Pizzi Mauro (Cadegliano-Viconago), Pizzocaro Andrea (Bardolino), Placenza Marilena (Quistello), Ploia Paola Roberta (Milano), Pluzarev Lijljana (Roncegno), Pocaterra Mirko (Budrio), Poggiani Maria Olivia (Saltara), Poggini Tiziano (Arezzo), Polastri Maria Grazia (Pieve Di Cento), Poletti Sonia (Alzate Brianza), Poliziani Dario (Lissone), Polizzi Giuseppe (Arsiè), Poloni Tiziana (Cafasse), Polotti Renzo (Rezzato), Pometi Claudia (Bologna), Pompili Giuseppina (Modena), Ponissa Amedeo (Monterenzio), Pons Roberto (Pinerolo), Ponziani Gabriele (Gallicano), Ponziano Mario (Modena), Porcu Rosa (Castelfranco Di Sotto), Porrozzi Carlo (Umbertide), Povoli Roberto (Spiazzo), Pozzoli Raffaella (Viganò), Praturlon Francesca (Caresanablot), Prearo Lino (Roana), Prefumo Matteo (Masone), Prevedello Giorgio Andrea (Crespano Del Grappa), Previati Stefania (Torino), Previderè Giorgio (Abbiategrasso), Prinzio Giovanna (Pinerolo), Prisco Elisa (Caglio), Procopio Giulia (Novalesa), Profeti Paolo (Montaione), Profili Giuseppe Simone (Chiusi), Pruzzo Mauro (Genova), Psimenos Angelos (Roma), Pugliese Caterina (Sasso Marconi), Puhalo Vesna (Brignano Gera D'Adda), Pulcinelli Giorgio (Civitavecchia), Puppi Valentina (Felino), Purifichi Brunella (Rosignano Marittimo), Putzolu Valeria (Costa Volpino), Qasem Ahmad Amedeo (Lerici), Quagliotti Eleonora (Volpiano), Quarta Luigi (Castri Di Lecce), Quarta Tommaso (Montefalcone Appennino), Quattri Enrico (Novara), Quattrone Antonio (Ostiano), Radicchi Luca (Roma), Radoani Matteo (Condino), Ragaglia Giampaolo (San Marcello), Raggi Giancarlo (Salò), Raina Daniele (Sezzadio), Raineri Chiara (Dogliani), Ramellini Luisa (Sartirana Lomellina), Ramello Cinzia (Alba), Ramuscello Sabina (San Michele Al Tagliamento), Randazzo Gerarmando (Firenzuola), Ranieri Sabatino (Collazzone), Ranzani Paola (Albairate), Raschella Giuseppa (Rocca D'Arazzo), Raspini Daniele (Figline Valdarno), Rata Laura (Giffoni Valle Piana), Ravarotto Jessica (Binasco), Ravazzoli Mauro (Esine), Ravazzolo Ester (Trento), Rech Roberto (Valdastico, Pedemonte), Reedy Susan (Brescia), Reineri Livia (Ciriè, San Maurizio Canavese), Remo Angelino (Pinerolo), Repetto Silvana (Lodi), Revelli Guido (Montanaro), Revetria Pietro Domenico (Garessio), Ribola Massimo (Broni), Riboni Vincenzo (Isola Vicentina), Ricagni Francesco (Frascaro), Riccarand Sergio (Lozio), Riccardi Anna (Montelupo Fiorentino), Ricci Camilla (Arezzo), Ricci Fabio (San Giovanni Rotondo), Ricci Giorgio (Villasanta), Riccioni Filippo (Lizzano In Belvedere), Ricutini Jessica (Firenze), Rillo Valentina (Rosignano Marittimo), Rimola Pasquale (Moglia), Rinaldi Liala (Montà), Risso Maura (Tiglieto), Riva Giuseppe (Tradate), Rizzato Antonio (Mirano, Conselve), Rizzelli Michele (Nociglia), Rizzi Guido (Taggia), Rizzi Nicola (Ravarino), Rizzo Tiziano (Crocetta Del Montello), Rizzo Vincenzo (Pontedera), Robazzi Adriano (Volta Mantovana, Castiglione Delle Stiviere), Robino Francesca (Genova), Rocca Mindreau Dante (Monfalcone), Rocchia Vincenzo (Castelfidardo), Rocco Lorella (Villata), Roccon Daniele (Pontelongo), Rodio Miriana Celeste (Lari), Roggia Maria Luisa (Parabiago), Rolfo Elio (Moncalieri, Torino), Rollandi Marco (Borghetto Di Vara), Romano Graziana (Parma), Roncato Stefano (Lozzo Atestino, Galzignano Terme), Ronchetti Emanuela (Modena), Ronchi Giuseppe (Sarezzo), Ronchini Monica (Vernasca), Ronchini Paolo (Fidenza), Ronconi Paolo (Morbegno), Rosin Rosanna (Caldogno), Rossato Domenico (Rubano), Rossazza Prin Marco (Graglia), Rosselli Cristina (Torino), Rossi Andrea (Bagnolo In Piano), Rossi Cristina (Ronco All'Adige), Rossi Daniele (Carpineti), Rossi Franceso (Montaquila), Rossi Franco (Montefalcone Appennino), Rossi Paolo (Barbarano Vicentino), Rossi Roberta (Magenta), Rossi Stefano (Arezzo), Rossini Riccardo (Ghedi), Rosti Virginio (Casei Gerola), Rota Alfredo (Lissone), Rota Michele (Valbondione), Rotta Andrea (Trieste), Roveda Susanna (Voghera), Rovini Gloria (Cecina), Rubatta Giovana (Cantù), Rubino Agnese (Modena), Rubino Giovanni (Malè), Rudella Lorenzo (Breganze), Ruffini Paola (Teglio), Ruffino Maria Grazia (Bosconero), Ruggeri Giulia (Tignale), Russo Mario (Torremaggiore), Rutigliano Valeria (Milano), Sabbatini Maura (Prato), Sabini Matteo (Trieste), Sabri Mohammad Mehdi (Crevalcore), Sacchelli Cristina (Castelleone), Sacchetto Alfredo (Oppeano), Sacchi Roberto (Treviglio), Saccol Guido (Vidor), Saccone Sara (Parma), Saffari Abolhassan (San Giovanni In Persiceto), Saggiorato Marco (Strigno), Sagnuolo Pasquale (Nago-Torbole), Salerno Francesco (Carugate), Salerno Maria Erica (Morciano Di Romagna), Salimbeni Isabella (Cingia De' Botti), Salvati Sergio (Ladispoli), Salvicchi Rodolfo (Foiano Della Chiana), Salvotelli Gianmarino (Riva Del Garda), Sambin Elena (Savona), Sambuco Patrizia (Mondolfo), Samorini Nadia (Conselice), Sampaoli Imelde (Sarsina), Sánchez Martín Sara (Como), Sanfelici Laura (San Giovanni In Croce), Sani Roberto (Cavaglià), Sanna Francesca (Minerbio), Sanna Lanfranco (Santo Stefano Di Magra), Santacaterina Silvia (Montecchio Precalcino), Santangelo Luigi (Napoli), Santesso Paolo (Livinallongo Del Col Di Lana), Santin Valentina (Agliè), Santolini Stefania (Russi), Santoro Elena (Seregno), Sanvito Fulvio (Monza), Sapoval Victor (Arco), Saracino Egidio (Bari), Sarnataro Nicola (Briosco), Sartori Michela (Casier), Sasanelli Giovanni (Noicattaro), Savona Valeria (Pianoro), Scapuzzo Alessandra (Gorla Minore), Scarabelli Riccardo (Rodengo-Saiano), Scarangella Antonio (Bari), Scarci Anna (Lazise, Castelnuovo Del Garda), Scarrone Giovanni (Villata), Scarsella Luca (Veroli), Scarselli Giampaolo (Sesto Fiorentino), Scartezzini Tiziana (Dro), Scaturro Melchiorre (Pieve Di Cento), Schianchi Poalo (Collecchio), Schiavano Salvatore (Casarano), Schiavinato Aurelio (Treviso), Schiavon Davide (Venezia), Schifino Nicola (Pavia), Schoepf Veruska (Spotorno), Sciumbata Valentina (Cesena), Scolaro Alessandro (Olginate), Sconosciuto Antonio (Massa, Carrara), Scordamaglia Lorenzo (Viareggio), Scornaienchi Bruna (Bocchigliero), Scranni Simone (Mercato Saraceno), Scudellari Roberta (Vobarno), Scusello Michele (Cavour), Secchi Simone (Trescore Balneario, Sale Marasino, Marone, Revere, Pegognaga), Segato Mauro Antonio (Santo Stino Di Livenza), Semeraro Vito (Priocca), Sensi Svaldo (Capannori), Serafini Paolo (Pietra Ligure, Vado Ligure), Serdyukovskaya Elena (Pianoro), Serena Alfredo (San Venanzo), Serena Errico (Baricella), Sergentu Rodica (Cavedine), Sergentu Smaranda Rodica (Nomi, Volano), Sergnese Giorgio (Bricherasio, Luserna San Giovanni), Sernelli Matteo (Bagno A Ripoli), Sero Arcangelo (Copparo), Serpi Sonia (Cermenate), Servadei Silvia (Faenza), Sesana Luigi (Civate), Settimio Rossana (Porto Recanati), Seves Giuseppina (Luserna San Giovanni), Sgreccia Vittorio (Senigallia), Sgrò Federica (Castenaso), Sgroni Walter (Lanzo D'Intelvi, Dizzasco, Pellio Intelvi), Sharham Ghyasaldin (Andria), Signani Francesca (Fivizzano), Signaroldi Alfredo (Piacenza), Sigurtà Francesco Donato (Soresina), Silvestri Milena (Riofreddo), Simone Francesco (Bisceglie), Simone Pasqualino (San Giovanni Rotondo), Simone Pasqualino Elio (Vieste), Simonetti Enrico (Altopascio), Simonini Gianluca (Pavullo Nel Frignano), Siniscalchi Alberto (Roma), Slawitz Antonio (Sorbolo), Soavi Francesco (Borgonovo Val Tidone), Soccini Giuseppina (Fiesco), Sodo Rosaria (Montefiascone), Sogari Fernando (Ginosa), Soregaroli Miria (Gottolengo), Sorlini Maria Luisa (Ospitaletto), Sorri Rolando (Firenze), Soumtcha Lengue Perrier Carmelle (Genova), Spadoni Roberto (Brugnato), Spadoni Silvia (Livorno), Spagnoli Andrea (Parma), Spagnoli Clara (Pontremoli, Bagnone), Spaziani Cesare (Fiuggi), Spidalieri Giuseppe (Cavour, Pinasca), Stanisci Laura (Ferrera Erbognone), Starcich Bruno (Parma), Stefani Gianpietro (Chiampo), Stefani Stefano (Romano D'Ezzelino), Stefanini Elena (Montechiarugolo), Stefanini Eugenia (Seravezza), Stella Gaetano (Caldogno), Stellato Raffaele (Lamon), Stenech Antonio (Rovereto), Stillitano Giuseppe (Sovere), Stocchero Giuliano (San Martino Dall'Argine, Guidizzolo), Stomati Dario (Lecce, Trepuzzi), Storti Andrea (Volta Mantovana), Strinati Flavio (Zibello), Stringari Alessandri Alessandro (Borgo A Mozzano), Studioso Eleonora Anna (Lipomo), Susta Mario Gianni (Como), Tabarroni Antonini Giorgio (Monte Giberto), Tachimiri Simona (Sondrio), Tagliani Giancarla (Desenzano Del Garda), Tagliani Giuseppe (Pontevico), Talarico Margherita (Cologne), Tallia Romina (Olcenengo), Tampanella Raffaele (Manduria), Tanghetti Paolo (Villa Carcina), Tarantino Antonio (Capurso), Tarantino Giovanni Stefano (Torino), Taranto Colombo Armando (Roma), Taroni Catia (Pianoro, Livorno, Aulla), Tarquinio Luana (Bologna), Tarsitani Gianfranco (Roma), Tassan Giada (Zagarolo), Tavoni Francesca (Bologna), Tayefeh Jafari Maryam (Manziana), Taylor Josephine (Magliano Alfieri), Tedde Antonio (Quartu Sant'Elena), Terreno Giovanni (Radda In Chianti), Terrezza Gino (Montepulciano), Terruli Ilaria (Parma), Terzi Agata (Marciana), Terzi Roberto (Graglia, Lessona, Occhieppo Inferiore, Mongrando), Tesini Novar Tesini Novar (Monte San Pietro), Tessari Emilio (Monteforte D'Alpone), Testa Michele (Chiampo), Testa Tommaso (Porretta Terme), Testasecca Barbara (Potenza Picena), Thakidil Anna (Pomarance), Tirelli Giuliano Cristian (Verolanuova), Tivisano Leonardo (Torremaggiore), Tizzoni Stefano (Ponte Dell'Olio), Todesco Domenica (Solagna), Tofani Nicoletta (Cividale Del Friuli), Toller Irene (Trento), Tolve Massimo (Tricarico), Toma Salvatore (Genova), Toma Sara (Portoferraio), Tomassoli Paola (Gubbio), Tomassoni Agnese (Falconara Marittima), Tomassoni Claudio (Montefano), Tombesi Fabio (Roma), Tomelini Michela (Torino), Tonarelli Graziella (Massa), Tondo Giuseppe (Cavallino), Toneatto Alberto (Fiesso D'Artico), Tonon Monica (Meduna Di Livenza), Tonon Tiziana (Vittorio Veneto, San Vendemiano), Topo Silvio (Ficulle), Torres Fulvio (Orbassano), Torsello Axsinia (Opera), Tortorolo Umberto (Genova), Toscano Diana (Bologna), Tosi Guido (Milano), Toussan Lavinia (Roma), Tovaglieri Maurizio (Gallarate), Tracanzan Assunta Chiara (Bassano Del Grappa), Travia Marco (Bologna), Travisani Martina (Ronco Briantino), Tritapepe Anna Maria (Villalago), Troilo Laura (Tivoli), Trolese Manola (Canal San Bovo), Trovè Andrea (Velletri), Tsoutsis Nikolaos (Modena), Tuccia Plinio (Cinisello Balsamo, Milano), Tumino Maria Luisa (Chiaravalle), Tundo Antonio (Bellaria-Igea Marina), Turchetto Eleonora (Sangano), Turci Fausto (Luino), Turci Marina (Carpi), Turi Vincenzo (Bari), Ugolini Luigi (Avigliana), Uliana Maria Aurora (Zero Branco), Ulinici Silvia (Brescia), Umeri Fulvio (Trieste), Vacca Alessandro (Torino), Vaccina Antonella Rita (Castelfranco Emilia), Valdonio Piergiorgio (Ottobiano), Valentini Rodolfo (Lecce, Maglie, San Pietro In Lama), Vallarino Luigi (Arenzano), Valori Virna (Corinaldo), Valterio Simonetta (Montescheno), Vanini Walter Emiliano (Piazza Brembana), Vanni Renato (Massa Marittima), Vannozzi Gioele (Grosseto), Vannucci Matteo Leone (Alessandria), Vanzi Nzey (Livorno), Vasilicò Marco Maria (Gorgonzola), Vassallo Orlando (Velletri), Vecchietti Marco (Rosasco), Vegezzi Luca (Pianello Val Tidone), Velimirovic Jelena (Ficulle), Vellone Roberto (Roccaforte Mondovì), Ventisette Grazia (Gardone Val Trompia), Ventura Maurizio Alberto (Travagliato), Venturi Patrizia (Crespellano), Venturini Donatella (San Bonifacio), Venturino Maurizio (Schio), Vercesi Andrea Mauro (Zoagli), Vercesi Maria Cristina (Farini), Vercesi Paolo (Belgioioso), Verdolin Dino (Arcole), Verna Annamaria (Zevio), Verni Lucia (Bagnone), Veronico Roberto (Altamura), Verre Monica (Chianciano Terme), Versace Paolo (Gorle), Vianello Donatella (Venezia), Vicenzi Mauro (Vallecrosia), Viganò Gianluigi (Scanzorosciate), Viganò Luca (Calvenzano), Vigliano Girando Sandra (Chivasso), Vigliotta Maria Teresa (Piedimonte San Germano), Vignola Marta (Vigone), Vignoli Susanna (Palazzuolo Sul Senio), Villa Maria (Lezzeno), Villa Tiziano (Bellagio), Violante Benedetto (Cuneo), Virgilio Leonardo (Concorezzo), Visconti Giovanna Maria (Dorno), Visigalli Andrea (Castelverde), Vispi Alberto (Novalesa), Vita Natale (Mesola), Vitali Nicoletta (Cotignola), Viti Franco (Pietrasanta), Vivaldi Nicoletta (Tortona), Volponi Maria Paola (Fermo), Vozza Carmen (Brindisi), Vulcano Vincenzo (Bologna), Wamba Dontsop Georges Josselin (Novellara), Yousef Hatem Mohamed (Monza), Zacchini Daniela (Viggiù), Zagami Annunciata (Como), Zajac Pavol (Caraglio), Zambarbieri Dario (Cervesina), Zambelli Riccardo (Cava Manara), Zambianchi Valentina (Varzi), Zanardi Luigi (Bologna), Zandonà Elisa (Solesino), Zanin Giorgio (Sarego), Zanini Lorenzo (Nave), Zanotti Luigi (Capriate San Gervasio), Zantedeschi Francesco (Bussolengo), Zappalà Giuseppe (Gravina Di Catania), Zappia Rosalba (Genova), Zappino Antonella (Moncrivello), Zavaritt Alessandro (Vertova, Stezzano), Zazzarini Tiziano (Tradate), Zeminian Piero Antonio (Borno), Zencher Andrea (Avio), Zimelli Mauro (Lumezzane, Concesio), Zingarelli Luciana (Bernalda), Zingaretti Giancarlo (Filottrano), Zoccarato Cinzia (Castelnuovo Calcea), Zorba Anjeza (Roma), Zortea Cinzia (Canal San Bovo), Zuccherelli Danilo (Grosseto), Zulli Luigi (Nemi), Zusso Giorgia (Maserada Sul Piave), Aversa Gabriele (Roma), Bajko Dalma Erzsébet (Roma), Cangelosi Denise (Palermo), Ciprelli Arianna (Roma), Corona Rosa (Roma), D'Angelo Roberta (Caserta), Della gatta Francesco (Roma), di Cicco Gianluca (Roma), Esposito Emanuele (Cassino), Fabrizi Elisa (Roma), Giorgi Giada (Roma), Guadagnoli Michela (Roma), Marino Mariapia (Roma), Meddi Virginia (Roma), Milana Gianluca (Roma), Selvaggi Ludovica (Palestrina), Tarantino Sofia (Roma), Terribili Chiara (Roma), Vuolo Giulia (Ischia)
